# Divergence patterns of genic copy number variation in natural populations of the house mouse (*Mus musculus domesticus*) reveal three conserved genes with major population-specific expansions

**DOI:** 10.1101/gr.187187.114

**Published:** 2015-08

**Authors:** Željka Pezer, Bettina Harr, Meike Teschke, Hiba Babiker, Diethard Tautz

**Affiliations:** 1 Max Planck Institute for Evolutionary Biology, 24306 Plön, Germany

## Abstract

Copy number variation represents a major source of genetic divergence, yet the evolutionary dynamics of genic copy number variation in natural populations during differentiation and adaptation remain unclear. We applied a read depth approach to genome resequencing data to detect copy number variants (CNVs) ≥1 kb in wild-caught mice belonging to four populations of *Mus musculus domesticus*. We complemented the bioinformatics analyses with experimental validation using droplet digital PCR. The specific focus of our analysis is CNVs that include complete genes, as these CNVs could be expected to contribute most directly to evolutionary divergence. In total, 1863 transcription units appear to be completely encompassed within CNVs in at least one individual when compared to the reference assembly. Further, 179 of these CNVs show population-specific copy number differences, and 325 are subject to complete deletion in multiple individuals. Among the most copy-number variable genes are three highly conserved genes that encode the splicing factor CWC22, the spindle protein SFI1, and the Holliday junction recognition protein HJURP. These genes exhibit population-specific expansion patterns that suggest involvement in local adaptations. We found that genes that overlap with large segmental duplications are generally more copy-number variable. These genes encode proteins that are relevant for environmental and behavioral interactions, such as vomeronasal and olfactory receptors, as well as major urinary proteins and several proteins of unknown function. The overall analysis shows that genic CNVs contribute more to population differentiation in mice than in humans and may promote and speed up population divergence.

Studying genetic variation in natural populations is key to understanding the evolutionary processes that lead to divergence. Genomic structural variation is a major contributor to genetic diversity in mammals. In a broad sense, structural variation encompasses genomic alterations of a wide size range, from small indels to whole chromosomes or even entire genome duplications, and refers to both unbalanced (i.e., duplications and deletions) and balanced structural differences (i.e., inversions and translocations). Recent efforts have focused on a particular form of structural variation typically known as copy number variation. Copy number variants (CNVs) are somewhat arbitrarily defined as DNA segments of over 50 bp in length that differ in copy number between two or more individuals (Alkan et al. 2011; Mills et al. 2011). According to some estimates, DNA regions that contain CNVs may account for >13% of the human genome, superseding the variance contributed by single-nucleotide polymorphisms (Stankiewicz and Lupski 2010). CNVs that are present in a population at frequencies higher than 1% are classified as copy number polymorphisms. The origin and maintenance of these variations have been associated with segmental duplications (SDs), which are ≥1 kb regions of high sequence identity that occur at more than one site in the genome (Eichler 2001; Scherer et al. 2007).

Given their size and abundance, many CNVs are likely to affect gene function and consequently influence organismal fitness (Schrider and Hahn 2010; Iskow et al. 2012). Indeed, some CNVs have been associated with complex disorders in humans, such as autism, schizophrenia, mental retardation, psoriasis, diabetes, and obesity (Henrichsen et al. 2009a; Stankiewicz and Lupski 2010; Girirajan et al. 2011). An increased frequency of CNVs has been demonstrated to correlate positively with cancer risk in healthy individuals (Shlien et al. 2008). Other CNVs have been found to be advantageous, and evidence for positively selected CNVs is accumulating (Iskow et al. 2012; Bryk and Tautz 2014). However, most CNVs appear to have mild or no phenotypic consequences, indicating that the majority of these variations may in fact be either neutral or at most slightly deleterious (Nguyen et al. 2008).

Although the polymorphisms that occur in CNVs are expected to impact evolutionary processes, systematic analyses in wild populations remain rare. Recent studies of natural populations of the three-spined stickleback suggested a special involvement of young genes in the generation of copy-number variation (Chain et al. 2014), as well as parallel selection for some CNV regions (Hirase et al. 2014). We analyze here copy number variation in natural populations of the house mouse, with a specific focus on full genes that are located within CNVs, as these genes could be particularly relevant for population differentiation and adaptation.

The wild population samples of *Mus musculus domesticus* that were used in our study have a very well-defined evolutionary history (Guénet and Bonhomme 2003; Cucchi et al. 2005; Rajabi-Maham et al. 2008; Hardouin et al. 2015) and are, therefore, particularly suitable for comparative evolutionary analyses. We have resequenced animals from populations from Germany and France that are genetically well differentiated (Ihle et al. 2006; Teschke et al. 2008; Staubach et al. 2012). These populations are derived from animals that colonized Western Europe ∼3000 yr ago and originated from populations in Iran (Cucchi et al. 2005; Rajabi-Maham et al. 2008; Hardouin et al. 2015). Accordingly, we use resequenced animals of this ancestral population for comparison. Further, we added to our analysis mice caught on Heligoland; these mice represent an island population with clear morphological differences from mainland animals (Zimmermann 1949; Reichstein and Vauk 1967). We reasoned that the known evolutionary relationships between these populations would provide an ideal framework for studying the role of CNVs in population divergence.

Among several available methodologies for structural variation detection, we selected a read-depth approach as the most appropriate strategy given our data set and study questions. We used the software tool CNVnator (Abyzov et al. 2011), which was suggested to be superior to other methods with respect to a number of properties, such as the accuracy of the copy number estimate, the precision of break point detection, and sensitivity and specificity (Duan et al. 2013). Our study revealed major differences in genic copy number in natural populations, which contribute extensively to genetic differentiation and ongoing population divergence.

## Results

Full genome resequencing data concerning individuals derived from four natural populations of the Western house mouse (*Mus musculus domesticus*) were used to assess copy number variation. Three populations were represented by eight individuals each (populations FRA from France, GER from Germany, and IRA from Iran), and one population was represented by three individuals (population HEL from the island Heligoland). All comparisons were made by mapping the reads (see Supplemental Table S1 for read mapping statistics) to the reference sequence (NCBI37/mm9) and calling CNVs using CNVnator (see Supplemental Text S1 for a discussion of the read-depth approach; Abyzov et al. 2011). CNVnator has been shown to be less reliable for detecting calls below 1 kb in length (Abyzov et al. 2011); hence, we do not consider such calls in our analyses and instead use the following criteria to classify CNVs (Supplemental Fig. S1A): “CNVs” are all duplications or deletions ≥1 kb; “genic CNVs” are calls that contain at least one whole transcription unit, based on the RefSeqGene database; and “CNV genes” are transcription units that are completely contained within genic CNVs. In addition, we use the term “CNV regions” (CNVRs) for genomic regions that include all partially or fully overlapping CNVs in any one of the analyzed animals. The borders of a CNVR are defined by the coordinates of the merged CNV calls across all individuals (Supplemental Fig. S1B).

### Digital PCR validation

To assess the accuracy of the computational inference of copy numbers based on read depth, we measured copy numbers experimentally using droplet digital PCR (ddPCR) at 23 different genomic regions. For the majority of the validated loci, the copy numbers determined using ddPCR correlated strongly with those predicted by CNVnator and were largely concordant in most individuals (Supplemental Fig. S2). We estimate that the false discovery rate of our call set is low (Supplemental Text S2) and consistent with estimates from human data (3%–20%) (Abyzov et al. 2011).

### Overall CNV comparisons

**Figure 1.**
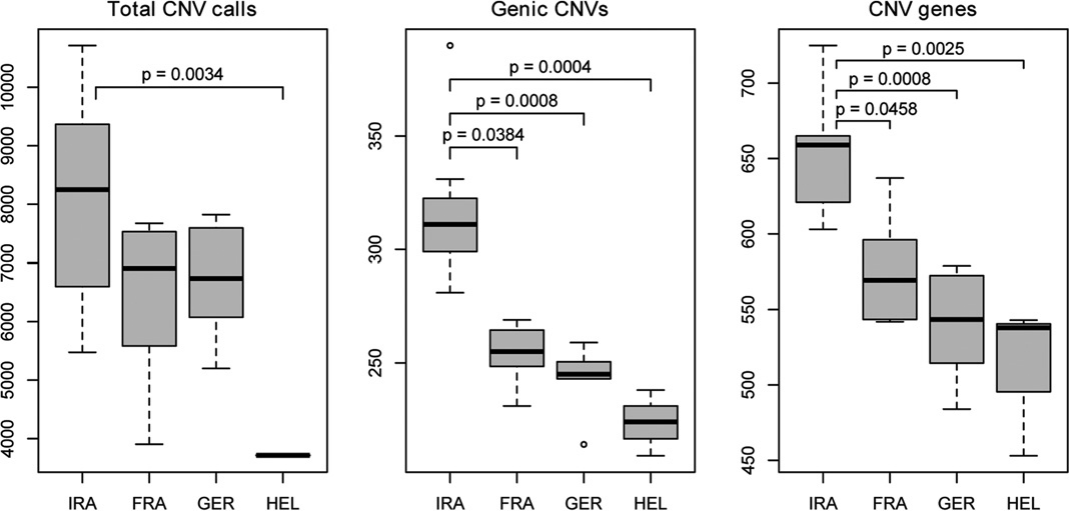
Number of detected CNVs. Distribution of CNV count classes for each population. The median of the population is indicated by the central line in a box, while the edges of the box represent the first and third quartiles. The Kruskal-Wallis test, followed by Dunn's post hoc test, was used to determine which differences were significant. *P* values were adjusted for multiple comparisons using the Bonferroni correction and are indicated only for pairs with significant differences (*P* < 0.05).

We first assessed the overall variation based on all CNV calls in all individuals. We found differences in the number of CNV calls per genome between individuals and populations (Fig. 1; Supplemental Table S1). The average number of detected CNVs was highest in population IRA (8078), intermediate in populations FRA (6453) and GER (6730), and lowest in population HEL (3714). The relatively lower number of CNVs found in population HEL can be largely explained by the reduced power to detect smaller CNVs and precise breakpoints in samples with a lower read depth (see Supplemental Text S3 for further explanation; Abyzov et al. 2011). The highest number of detected genic CNVs and CNV genes in population IRA likely reflects the larger effective population size (Fig. 1; central and right panels). We also assessed singletons and deletions (Supplemental Text 4) and found that ∼2% of the genome may be subject to deletions/insertions when comparing any wild mouse sample with the reference genome.

The presence and absence patterns of CNV calls between individuals show the expected grouping of populations (i.e., individuals within populations are more similar to each other than individuals between populations) (Supplemental Text S5; Supplemental Fig. S5). When compared with previously published data concerning structural variation in inbred mouse strains (Keane et al. 2011; Wong et al. 2012), we found that our wild mice samples are most similar to the wild-derived strain of *M. m. domesticus* (WSB/EiJ) and the laboratory strain FVB/NJ, based on the number of overlapping CNVs (Supplemental Text S5; Supplemental Fig. S6).

### CNV frequency and segmental duplications

Associations between CNV polymorphisms and SDs have been described for humans and for inbred mouse strains (Sebat et al. 2004; Sharp et al. 2005; Egan et al. 2007; She et al. 2008). Therefore, we investigated whether this finding also holds true for wild mouse populations. We focused on SDs longer than 10 kb, as these SDs are more likely to cause meiotic misalignment and aberrant recombination (Stankiewicz and Lupski 2002; Sharp et al. 2005). Given that CNV calling can be distorted due to read mismapping, we tested the performance of CNVnator in regions with highly similar sequences and found no major concerns related to misalignment in our data set (Supplemental Text S6).

**Figure 2.**
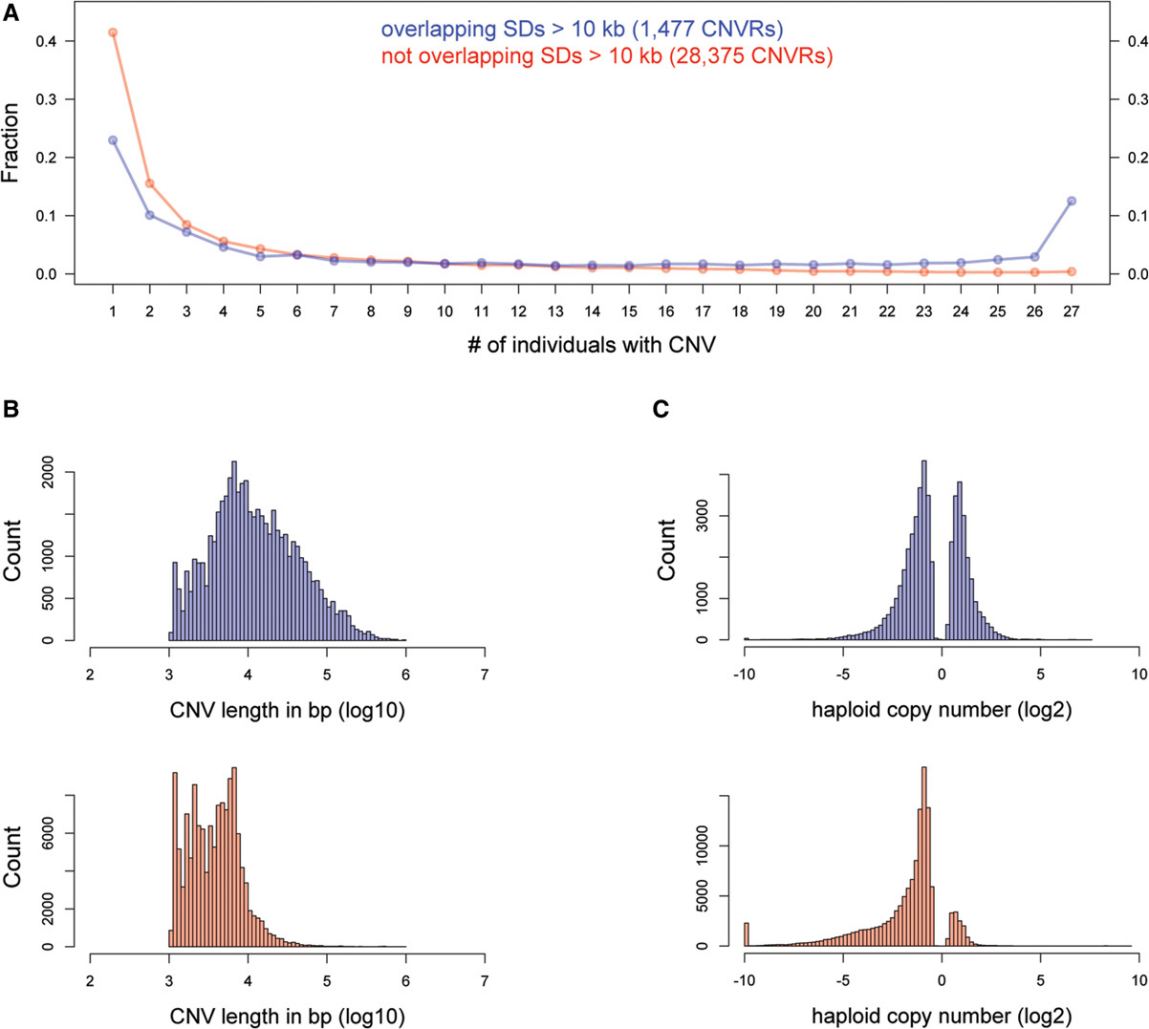
CNVRs that overlap with large SDs are present in multiple individuals. Overlapping calls from all individuals were merged into CNVRs and analyzed separately based on their intersection with SDs >10 kb. The number of individuals with CNV calls within each CNVR was counted (*A*). In total, we identified 1477 unique CNVRs that overlapped with SDs (blue) and 28,375 CNVRs that did not overlap with SDs (red). The graph shows the frequencies of CNVR presence across all samples. The length (*B*) and copy number (*C*) distributions of CNVs that overlap with genes differ significantly between the two data sets (KS test, *P* < 2.2 × 10^−16^). In the histograms shown in *C*, the bin with −10 values corresponds to events with absolute deletion; to ensure that all data were log_2_-transformable, these values were converted to a value of 0.001.

To compare loci across all individuals, we used CNVRs and partitioned those CNVRs into two sets: CNVRs that intersect with annotated SDs in the reference genome and CNVRs that do not intersect with annotated SDs. Within each of these sets, we counted the number of animals with actual CNV call(s) present (Fig. 2A). The two sets had significantly different distributions (Kolmogorov–Smirnov [KS] test; *P* < 2.2 × 10^−16^). In the set that does not overlap with SDs, the majority of CNVRs were found in only a few animals (over 40% were found exclusively in one animal, and ∼25% were found in two or three animals), and <1% of all CNVRs were shared among all 27 individuals. This finding cannot be ascribed to the CNVR size distribution (Supplemental Text S7). In the set that does overlap with SDs, we found that ∼13% of CNV regions are shared by all animals, and 20% are shared by at least 24 animals, whereas ∼23% are present exclusively in one individual; however, this set contains a total of 340 CNVs, or an average of 13 CNVs per individual, as opposed to nearly 12,000 CNVs in the nonoverlapping SDs set. The differences between the two sets were even more pronounced when we considered only CNVRs that overlap with genes (Supplemental Fig. S9).

The CNV calls within CNVRs that do not overlap with SDs were significantly smaller (median size 3.8 kb, average 5.5 kb) than those within CNVRs that overlap with SDs (median size 10.7 kb, average 28.5 kb) (Fig. 2B). The former group also had a lower average copy number than the latter group (0.67 versus 1.27 haploid copies) (Fig. 2C) and was generally depleted of duplications.

We found major differences in gene ontology (GO) term enrichment between the two sets. CNVRs that overlap with SDs are dominated by vomeronasal receptors and olfactory genes and are enriched for processes such as the sensory perception of taste, immune response, and G-protein coupled receptor signaling pathway (Supplemental Table S2). The association of CNVs with gene families involved in these processes has also been reported in other species and in inbred mouse strains (Perry et al. 2006; Cutler et al. 2007; Guryev et al. 2008; She et al. 2008; Chen et al. 2009, 2012; Doan et al. 2012). Genes in CNVRs that do not overlap with large SDs showed enrichment for terms related to a much broader spectrum of biological processes, such as protein modification, signaling, and ion transport (Supplemental Table S3). These categories contain genes that play a variety of roles in the regulation of development, cellular growth, and differentiation and include many genes that encode protein kinases, phosphatases, oncogenes, voltage-gated channels, and neurologically functioning genes. The size of genes involved in brain function has been demonstrated to be a significant confounder when performing gene set enrichment analyses on CNVs and to cause spurious findings (Raychaudhuri et al. 2010). By performing permutations of CNVRs across the genome, we show that despite their relatively larger size, this is not the case for genes associated with brain functions in our data set (Supplemental Text S8).

### Variation in gene content

Functional dosage effects could have direct impacts on adaptive population differentiation. Therefore, we sought to identify CNVs that affect whole genes, namely genic CNVs and CNV genes within them (see Supplemental Fig. S1A for definition). By using CNVnator's “genotype” option, we determined the copy number for the CNV transcription units that are duplicated or deleted over their whole length in at least one animal when compared to the reference genome. We found a total of 1863 such units (see Supplemental Text S9 for comments on genotyping accuracy and Supplemental Table S4 for overall results).

**Figure 3.**
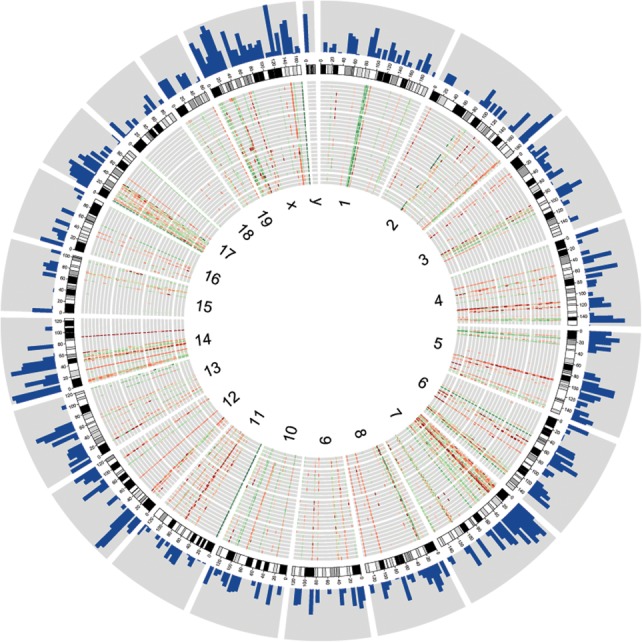
Genome-wide distribution of CNV genes. CNV genes are represented in individual tracks as heat maps, where red bars depict deletions and green bars depict duplications with respect to the reference assembly. Darker shades illustrate higher degrees of copy number change. The data tracks are organized concentrically from the *outer* circle to the *inner* circle: histograms of SD (>10 kb) density per 5-Mb window (log_2_ scale); chromosome ideograms; eight individuals of each French, German, and Iranian population; three Heligoland mice; and chromosome numbers. The graph was plotted using Circos (Krzywinski et al. 2009).

CNV genes are not evenly distributed across the genome but are rather clustered along certain chromosomal regions (Fig. 3). Such CNV “hotspots” have been shown to co-occur with SDs, and similar genomic distribution patterns have been reported for great apes and inbred mouse strains (Sharp et al. 2005; Perry et al. 2006; Egan et al. 2007; Graubert et al. 2007; Gazave et al. 2011). A total of 68% of the CNV genes in our data set overlapped with large SDs in comparison to <8% of the total RefSeq gene set (hypergeometric test; *P* < 5 × 10^−115^), indicating strong association between the two. An analysis of gene function categories revealed enrichment in processes related to sensory perception and immune response (Supplemental Table S5), and approximately one quarter of genes were annotated as olfactory and vomeronasal receptors.

A total of 65% of the identified CNV genes (1218) show deletions in at least one of the animals, and the majority of these loci (1020) show the deleted allele in multiple individuals. Accordingly, of the 1091 CNV genes on autosomes, 444 are homozygous for the deletion allele in at least one individual and 325 are homozygous for the deletion allele in multiple animals. Of the 127 CNV genes on the X Chromosome, 122 have a complete deletion in at least one male or one of the two females, and 99 loci are fully deleted in more than one individual. We calculated the frequency of the deletion allele for each of the 1218 loci based on the number of animals with copy numbers zero and one, taking into account the hemizygous state of the X Chromosome in males. The average frequency was 25%, and we identified 215 loci with a frequency of 50% or higher (Supplemental Table S6). Most of these genes show no further amplification in any population; however, some of these genes exist in more than two copies per genome. For these genes, it is impossible to infer with absolute certainty the allelic configuration that constitutes the estimated total number of copies. For instance, although it is more likely that a copy number of three represents a CN1:CN2 configuration than a CN0:CN3 configuration (the latter would require more than one mutational event), we did not consider these cases in our calculations. Therefore, our results represent lower bound estimates of the actual frequencies and indicate segregation of the deletion allele in the population. In HEL samples, substantially more autosomal genes (71) appear to be completely absent (i.e., they have homozygous deletions in all of the analyzed animals within the population) in comparison to the GER, IRA, and FRA samples (one, six, and 16 genes, respectively). Although these numbers are expected to depend on the number of animals analyzed, we demonstrate that the number of lost genes in the HEL population is likely to be significantly larger than in mainland populations (Supplemental Text S10).

In our data set, 26 genes distributed in 14 locations in the reference genome have, on average, 10 or more copies (i.e., they can be considered to be high copy number genes). These genes include ribosomal RNA genes, as well as genes that are annotated as single copy in the reference genome, such as *Cwc22*, *Hjurp*, and *Sfi1* (Table 1). The raw read-depth signal at CNVs encompassing these genes suggests that the CNV breakpoints are at approximately the same location in all individuals (Supplemental Fig. S13).

**Table 1.**
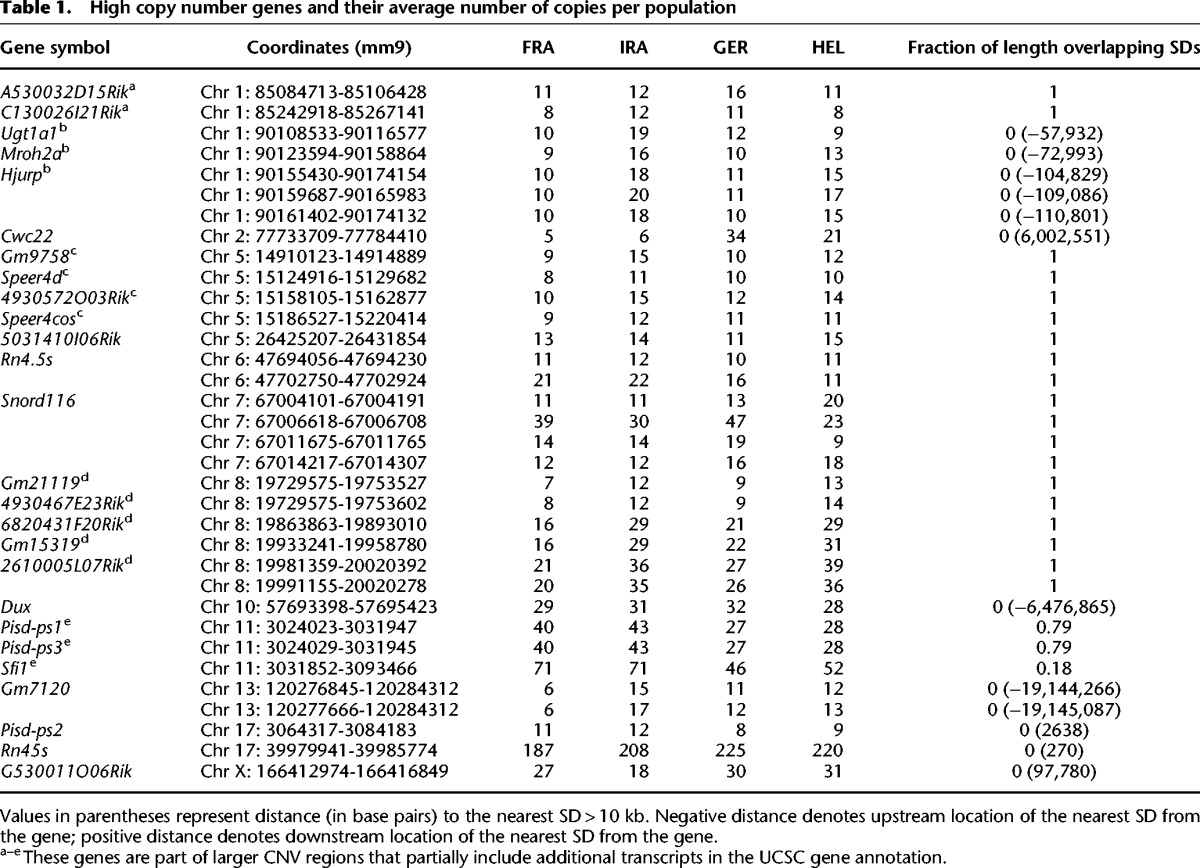
High copy number genes and their average number of copies per population

### Population differentiation of CNV genes

**Figure 4.**
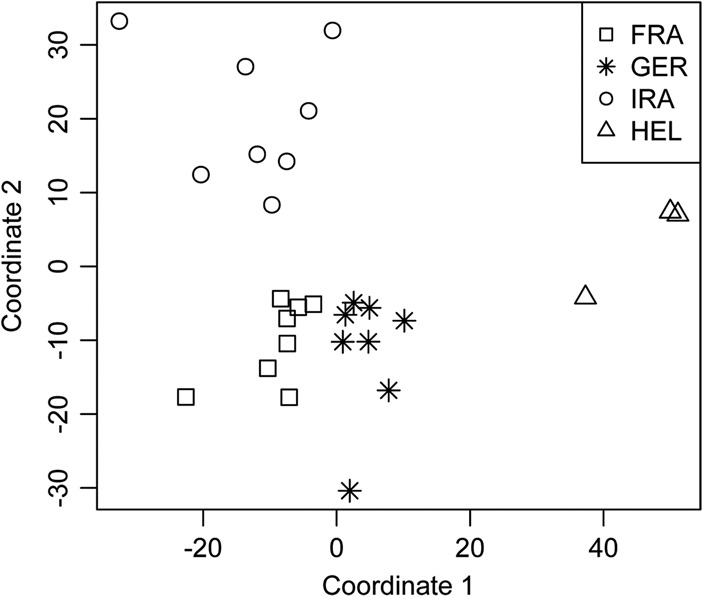
Two-dimensional representation of nonmetric multidimensional scaling (MDS). The analysis is based on the dissimilarity matrix generated by calculating the Euclidian distance between each of the possible pairwise comparisons of 27 individuals across 1863 CNV genes. Each dot represents one individual.

To determine whether the overall variation in CNV gene content captures the known evolutionary relationships among populations (Guénet and Bonhomme 2003; Cucchi et al. 2005; Rajabi-Maham et al. 2008; Hardouin et al. 2015), we performed a multidimensional scaling analysis (MDS) on the Euclidean distance dissimilarity matrix calculated from standardized copy numbers of CNV genes. The clear separation into distinct groups that correspond to the four populations (Fig. 4) shows patterns of relationship and variance that are similar to those observed in the analysis of all CNVs (Supplemental Fig. S5). Both comparisons confirm that the closest relationship exists between GER and FRA mice and that the largest variation occurs within the IRA population. While the HEL mice are the most different in comparison to the mice in all of the other populations, those mice are still closer to the GER population than to the other two populations, which is consistent with the geographical proximity of those populations.

To identify genes whose copy numbers show signatures of population differentiation, we applied the *V*_ST_ statistic (Redon et al. 2006). We calculated *V*_ST_ for each CNV gene in each pairwise comparison (Supplemental Table S7). The average *V*_ST_ values across all genes were 0.14, 0.11, and 0.15 for the FRA-IRA, FRA-GER, and IRA-GER comparisons, respectively. The higher average *V*_ST_ values obtained in the comparisons with IRA are consistent with the higher degree of differentiation between ancestral and derived populations. Even larger values were observed in all comparisons with HEL mice (between 0.24 and 0.25 on average), which cannot simply be ascribed to the small sample size (Supplemental Text S11). When compared to human populations (Redon et al. 2006), the overall *V*_ST_ values in our mouse data set are substantially higher, suggesting stronger population differentiation in mice. Among the genes with the highest *V*_ST_ values in pairwise population comparisons are those known to occur in gene clusters, such as the serpin proteinase inhibitors, the major urinary protein genes, vomeronasal and olfactory receptors, and amylase genes, as well as several predicted genes of unknown function (Supplemental Table S7).

Pairwise comparisons also revealed genes with particularly large differences in average copy number between populations (Supplemental Text S12). An extreme variation is seen for the *Cwc22* gene; in our data set, this gene ranges from 2 to 83 copies and is, on average, the most amplified gene in the German population (Supplemental Fig. S15B). In addition to the outliers, a considerable fraction of genes differed in their average copy number (Supplemental Fig. S15A). An ANOVA analysis yielded a total of 179 genes (Supplemental Table S8) with significant differences between at least two populations (one-way ANOVA, *F*_(2,21)_ > 5.77, *P* < 0.05). HEL mice were excluded due to the small sample size. Among the genes annotated as singleton genes in the reference genome, *Sfi1* showed unexpectedly high variation and population divergence (one-way ANOVA, *F*_(2,21)_ = 53.57, *P* < 0.0001 after FDR).

### Extended analysis of *Cwc22*, *Hjurp*, and *Sfi1*

**Figure 5.**
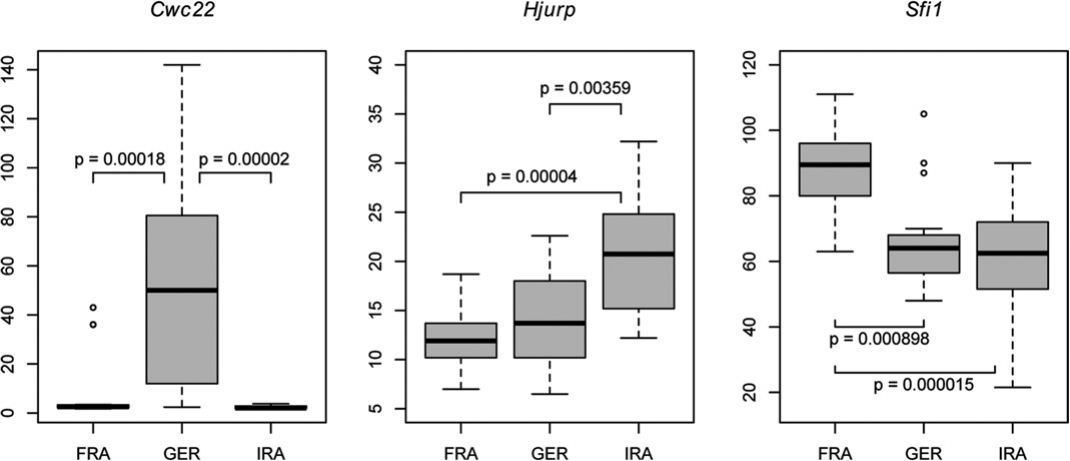
Extended population survey for extreme CNV loci. ddPCR was used to measure the copy number of *Cwc22*, *Hjurp*, and *Sfi1* in animals belonging to the French (*n* = 16), German (*n* = 15), and Iranian (*n* = 16) populations. The distributions of their copy numbers per population is shown as a boxplot. To detect differences between the populations, ANOVA was performed, followed by Tukey's HSD. Only significant *P* values (*P*-adj < 0.05) are shown.

To study the polymorphism of extreme outlier loci in more depth, we evaluated the copy numbers of *Cwc22*, *Hjurp*, and *Sfi1* in additional unrelated individuals belonging to the three mainland populations. We confirmed the high copy number profile of all three genes using ddPCR (Fig. 5). Interestingly, each CNV gene shows a significant major expansion in one of the populations. *Cwc22* is highly expanded in the German population; *Hjurp* has, on average, nearly twice as many copies in the Iranian population; and *Sfi1* has 50% more copies in the French population. For *Cwc22* and *Sfi1*, these data are inconsistent with the overall trend of CNV divergence between these populations (Fig. 4). Hence, it appears likely that these differences occurred after the population split and that these genes could have been driven to a higher copy number by positive selection.

## Discussion

Substantial copy number variation in inbred mouse strains has been noted previously (Cutler et al. 2007; Egan et al. 2007; Graubert et al. 2007; Keane et al. 2014). Structural variations are known to accumulate during the inbreeding process (Katju and Bergthorsson 2014); however, the extent to which these variations contribute to divergence in natural mouse populations remained unknown until now. We found that CNVs show stronger population stratification in wild mice than in humans (Jakobsson et al. 2008; Itsara et al. 2009) and contribute substantially to genetic differences. We identified several hundred genes that are fully deleted in one or more animals, supporting a process of continuous gene turnover in natural populations (Neme and Tautz 2014). On the other hand, the most drastic copy number variations include highly conserved genes with major roles in essential cellular processes, indicating that even these genes are subject to rapid evolutionary modification within and between populations.

The overall CNV divergence patterns observed in mainland mice are consistent with the known evolutionary relationships between these populations, implying a more or less constant accumulation of differences over time. Such progressive divergence is predicted by the neutral model of evolution, which assumes that mutations, drift, and purifying selection shape molecular evolution, while adaptive mutations are expected to be rare (Kimura 1983). Our results suggest that most CNVs evolve according to such a neutral model, whereas the identified outlier loci are candidate genes for specific adaptations.

### Association with segmental duplications

It has been suggested that nonallelic homologous recombination could drive copy number changes in regions that overlap with SDs (Stankiewicz and Lupski 2002; She et al. 2008). The genomic distribution of CNVs and the strong association of CNVs with SDs in the mouse populations support this idea. Opposing evidence came from a recent study, which indicated that SD regions are recombination deserts (Liu et al. 2014). However, the founder strains used for the generation of the Collaborative Cross mice analyzed in this study include other subspecies, which may result in atypical recombination patterns. Moreover, mitotic recombination may occur at a much higher rate than meiotic recombination in tandemly repetitive regions (Schlötterer and Tautz 1994), resulting in higher copy number variation in these regions. Accordingly, we found that CNVs that overlap with SDs are much more variable between individuals. A large number of CNVs in regions that do not overlap with large SDs are found only in single individuals, concordant with previous findings in humans and apes (Redon et al. 2006; Wong et al. 2007; Gazave et al. 2011). The two categories of CNVs show major functional differences. CNVs that overlap with large SDs are enriched for nonessential, environmentally responsive genes, which are proposed to be under less stringent evolutionary constraints (Nguyen et al. 2008), while CNVs that do not overlap with large SDs are enriched for genes that have been linked to Mendelian diseases and are similarly overrepresented in CNVs outside of SDs in humans (Cooper et al. 2007; Nguyen et al. 2008). The latter CNVs are much smaller in length, amplitude, and frequency, suggesting stronger selective constraints against structural variation in genes whose disruption might lead to disease.

### Amplifications versus deletions

Structural variations include both amplifications and deletions of genomic regions, which are processes with potentially different biological and evolutionary consequences. The amplification of whole genes can have a direct impact on gene expression by changing gene dosage (Perry et al. 2007; Watkins-Chow and Pavan 2008; Henrichsen et al. 2009b; Orozco et al. 2009; Stingele et al. 2012; Katju and Bergthorsson 2014). If a copy number change is beneficial, it will be favored by selection and retained more frequently in a population, resulting in average differences in gene copy number between populations. By investigating these differences, several CNV genes have been found to be under positive selection in human populations, such as the amylase and *CCL3L1* genes (Gonzalez et al. 2005; Perry et al. 2007). We identified 13 genes in our data set whose copy number differences between populations are similarly compatible with signatures of positive selection. Seven of these genes are amplified in the derived populations (FRA and GER), suggesting possible adaptation to a new environment after colonization.

In contrast, deletions of genes are unlikely to be adaptive. However, as part of their life cycle (Neme and Tautz 2014), genes can be lost when their contribution to fitness is small and when the population size is limited. Indeed, we found that substantially more genes appear to be completely deleted in Heligoland mice than in mainland populations, which could reflect the small effective population size of islander mice. A large number of genes in the human genome also show deletion polymorphism (Conrad et al. 2006; Feuk et al. 2006; McCarroll et al. 2006). Most of those genes were identified as members of gene families, and this finding has been used to explain the higher tolerance for null alleles (Feuk et al. 2006). Many genes with deletion alleles in our data set are also specific members of large gene families or transcripts of unknown function (Supplemental Table S6).

### CNV genes of special interest

The gene with the largest range of copy number differences between populations in our sample is *Cwc22*. This gene encodes a broadly conserved spliceosome-associated protein, which has been shown to be indispensable for pre-mRNA splicing in humans (Steckelberg et al. 2012) and is associated with nonsense-mediated decay (Alexandrov et al. 2012). The high copy number variation of *Cwc22* was also shown to correlate with variability in H3K4me3 methylation marks and the expression of the gene in the livers of wild mice (Börsch-Haubold et al. 2014). Intriguingly, an expansion of *Cwc22* has been implicated in a segregation distortion effect in heterozygous female mice (Didion et al. 2015). Therefore, an initial increase in copy number could lead to the rapid propagation of even more copies within a population when the segregation distortion favors its own transmission of alleles with a larger number of copies. However, it remains unclear which molecular processes would cause this phenomenon and why *Cwc22* expansion has only been found in one population to date (our study) and in association with particular genetic backgrounds (Didion et al. 2015).

Similar questions apply to *Sfi1* and *Hjurp*. *Sfi1* encodes a conserved centrin-binding protein that is associated with spindle assembly (Kilmartin 2003) and exhibits high variation and differentiation between populations. This gene has been estimated to exist in 20–30 copies in the genomes of inbred mouse strains (Quinlan et al. 2010), and we found 20–110 copies in wild mice (Fig. 5; Supplemental Table S4). *Hjurp* mediates the centromere-specific assembly of CENP-A nucleosomes, contributing to high-fidelity chromosome segregation during cell division (Stellfox et al. 2013). The misregulation of *Hjurp* has been shown to affect chromosome stability in yeast and human cells (Mishra et al. 2011), and expression levels in human cells influence senescence (Heo et al. 2013). Similarly to *Cwc22*, *Hjurp* was also found to have variable expression and methylation marks in experiments with wild mice (Börsch-Haubold et al. 2014).

High differentiation between populations is also found in the amylase genes *Amy2a5* and *Amy2a2*. Compared to the reference genome, we found additional copies in FRA mice and deletions in IRA and HEL mice (Supplemental Table S9). Duplications of the amylase genes have been suggested to be a result of adaptation to a starch-rich diet in human populations (Perry et al. 2007) and dogs (Axelsson et al. 2013). However, the genes analyzed in these studies (i.e., the salivary gene *Amy1* and the pancreatic gene *Amy2b*) are not part of the duplications in our study, and we are currently investigating alternative interpretations for the pattern observed in this region (M Linnenbrink and D Tautz, in prep.).

The major urinary protein (*Mup*) cluster exhibits striking differentiation along its whole length, including many genes, and in all population comparisons (Supplemental Tables S7, S10; Supplemental Fig. S16). These genes were previously identified as copy-number variable among inbred mouse strains (She et al. 2008). MUPs are proteins that are excreted abundantly in mouse urine and are thought to regulate social behaviors, such as aggression (Chamero et al. 2007) or mating (Marchlewska-Koj et al. 2000; More 2006), by acting as pheromones. The *Mup* gene families have been demonstrated to exhibit remarkable lineage specificity, which is consistent with a role in species-specific communication (Logan et al. 2008). In addition, MUPs have the potential to be used for individual discrimination, as shown in wild mice, where unique combinations of MUPs expressed in urine serve as a personal olfactory fingerprint (Hurst et al. 2001). Such recognition is crucial for many aspects of behavior, including mate choice, inbreeding avoidance, maternal bonding, and territory establishment. In addition to the individual and lineage specificity of the mouse *Mup* cluster, our data suggest population specificity in the form of CNV patterns. If this level of recognition controls mate choice, it may lead to preferential mating with individuals from the same population and ultimately result in population divergence. Indeed, we previously found evidence for assortative mating in the FRA and GER populations; given the recent divergence of those populations, we proposed the rapid evolution of the recognition system (Montero et al. 2013).

The *V*_ST_ (Supplemental Table S7) and the ANOVA analysis (Supplemental Table S8) provide additional candidate genes that could contribute to population differentiation. However, it is currently impossible to say how much of this differentiation may have been due to positive selection versus neutral drift. Neutral processes can result in population divergence, and such processes can contribute to the splitting of populations and eventually to speciation by affecting genes relevant for environmental or behavioral interactions. Because different gene families and chromosomal regions (such as those that overlap with SDs) are expected to show different structural mutation rates, it is not possible to simulate a simple whole-genome null model against which selection models could be tested. Therefore, we expect that evidence for the involvement of positive selection will have to be obtained on a gene-by-gene basis. Our study provides suitable candidate genes for such further analysis.

## Methods

### DNA samples and sequencing

Whole-genome sequences of 27 mice were analyzed; 25 of the analyzed animals were males, and two were females (samples HG_06 and HG_13). The mice were caught in the wild following the sampling scheme, which ensures that individuals are not related to each other and represent local populations (described in Ihle et al. 2006). Eight mice were obtained from different farms from each of the three mainland regions: Cologne-Bonn (Germany), Central Massif (France), and Ahvaz (Iran) and were first generation offspring of the wild caught mice. Each mating was set up with one male and female that were caught at the same farm to ensure that the analyzed offspring represent the natural genetic variation of wild caught animals. Three individuals were sampled on Heligoland Island (Germany, North Sea) and were not bred further. DNA was extracted from the tails (mainland mice) and livers (Heligoland mice) of the mice. A paired-end DNA library with an insert size of ∼230 bp was prepared for each genome by the sequencing center (Cologne Center for Genomics, University of Cologne, Germany), according to the standard Illumina TruSeq protocol for sequencing on a HiSeq 2000 (Illumina). The resulting libraries were tagged, pooled, and sequenced using a paired-end cluster generation kit on an Illumina HiSeq 2000 (2 × 100 bp). The reads were mapped onto the mouse reference genome (mm9) using BWA (Li and Durbin 2009). PCR duplicates were removed using SAMtools (Li et al. 2009). The final average coverage was ∼20× for the mainland populations and 10× for the Heligoland samples (see Supplemental Table S1 for read mapping statistics).

Additional unrelated individuals from the three mainland populations were collected as described above and used for validation by ddPCR as independent sample set; this set included 16 mice each from the French and Iranian populations and 15 mice from the German population.

### Genomic data sets

All reference data were obtained from the University of California Santa Cruz (UCSC) Genome Browser (http://genome.ucsc.edu). The genomic sequence of *Mus musculus domesticus* from the NCBI Build 37 assembly (mm9) was used as a reference for read mapping and CNV calling. The coordinates of segmental duplications were downloaded for the same assembly (737,482 in total), and only those that defined sequences longer than 10 kb were retained for further analysis (256,081). The RefSeq gene set (30,823) was reduced to a nonredundant list of coordinates that corresponded to 26,756 unique transcription units (defined by transcription start and end sites).

### Droplet digital PCR validation

To validate the CNVs detected by CNVnator, PrimeTime qPCR assays (Integrated DNA Technologies) were used in duplex reactions to measure the copy number in altered regions (target) relative to a control region (reference) with an invariant copy number across all 27 individuals. For the control region in all reactions, we chose a fragment of the *Tert* gene, which was present in two copies per diploid and showed no CNV calls in any part of the gene for all samples. The relative copy numbers were determined using the QX100 Droplet Digital PCR System (Bio-Rad). Primers and probes (Supplemental Table S12) were designed using the Primer3 software (http://bioinfo.ut.ee/primer3-0.4.0) and checked for specificity using NCBI's Primer-BLAST (http://www.ncbi.nlm.nih.gov/tools/primer-blast). The probes for target genes were labeled with FAM, and the probes for the control region were labeled with HEX. All target assays were chosen to be compatible with the *Tert* reference assay, and an initial PCR was performed for each reaction to determine the optimal annealing temperature. The DNA samples were digested with the BamHI restriction endonuclease (New England BioLabs) at a concentration of 100 ng of DNA per 30-μL reaction, using 5 units of enzyme per 1 μg of DNA. Restriction digestions were incubated for 45 min at 37°C, followed by 20 min of inactivation at 65°C. Assays *Luzp4* and *Gm21671* were also digested with MseI to prevent the amplification of nonspecific targets (Supplemental Text S2). The digested DNA samples were added to the Bio-Rad 2× ddPCR supermix at concentrations of 3–5 ng per 20 μL ddPCR reaction. The assay primers and probes were present at final concentrations of 900 nM and 250 nM, respectively. The reaction mixtures were converted into droplets, which were then subjected to amplification, as follows: one cycle at 95°C for 10 min, 40 cycles at 94°C for 30 sec, and 60°C or 61°C for 1 min, and 98°C for 10 min, with a ramp speed of 2.5°C/sec. After PCR, the reactions were loaded onto the QX100 Droplet Digital reader, and analysis was performed using Bio-Rad's Quantasoft software.

### CNV detection

The CNVnator software was used to predict CNV calls relative to the mm9 reference assembly (Abyzov et al. 2011). The optimal bin size for each individual was chosen according to the authors’ recommendations, such that the ratio of the average read-depth signal to its standard deviation was between 4 and 5. The bin size ranged from 150 to 400 bp and was inversely proportional to the genome coverage. Calls intersecting annotated gaps in the reference genome were not considered. Approximately 4%–18% of the calls in mainland samples were smaller than 1 kb, whereas all detected events in the Heligoland samples were longer than 1 kb. This difference in CNV size distribution is expected given the lower coverage in Heligoland mice and the consequently reduced power to detect small CNVs. Hence, to avoid misinterpreting the results due to this bias, events smaller than 1 kb were not considered in our analyses.

For comparisons of CNVs among multiple individuals, we merged all overlapping calls across individuals into unique CNVRs. All analyses that required call intersecting or merging were performed using BEDTools (Quinlan and Hall 2010).

For gene copy number comparisons, CNV calls were intersected with the RefSeq gene set, and only those coordinates that were entirely confined to the predicted CNVs were retained. We refer to this set as “CNV genes.” Note that this set includes records annotated as pseudogenes and predicted genes. We did not remove these records for two reasons. First, the RefSeqGene database is a curated collection of nonredundant, well-supported genomic sequences that resemble genes in structure. Second, annotated “pseudogenes” can be functional (Pei et al. 2012) and are known to be highly polymorphic in copy number (Ewing et al. 2013; Schrider et al. 2013). Therefore, the potential influence of these “pseudogenes” on fitness and population dynamics makes them functionally and evolutionarily relevant.

### Copy number estimates

To compare gene copy number (CN) between individuals, we used CNVnator to determine the average CN across the gene length (“-genotype” option). This approach was necessary due to the complexity of CNV calls in many genes. For example, different degrees of both deletions and duplications can be found within the same gene. Furthermore, each animal showed copy number variation in only a subset of these genes, and sometimes a gene would only partially overlap with the CNV call.

### GO analysis

An analysis of gene ontology term enrichment was performed across all samples. The CNV calls from all individuals were merged into CNVRs, which were then partitioned according to their overlap with large SDs (>10 kb). This process resulted in 49,242 CNVs (1477 CNVRs) in the SD-overlapping set and 131,983 CNVs (28,375 CNVRs) within the SD-nonoverlapping set. The gene content in each set was analyzed using GOrilla (Eden et al. 2009), with the whole RefSeq gene set as background. We focused on GO categories associated with “Biological process” and considered significant only those for which FDR was <5%.

### Population differentiation

To identify CNV genes with high differentiation between populations, the *V*_ST_ statistic was applied (Redon et al. 2006). For each pairwise population comparison, *V*_ST_ was calculated for every CNV gene as *V*_ST_ = (*V*_T_–*V*_S_)/*V*_T_, where *V*_T_ is the total variance in CN between the two populations and *V*_S_ is the average of the variance within each single population, weighted for its sample size.

To detect differences in CN frequency between genes in the FRA, GER, and IRA populations, we applied ANOVA to the whole CNV gene set. Heligoland mice were excluded from the analysis because the sample size was too small and unequal to the other three populations. After correcting for multiple testing by FDR at 5%, 392 genes had a *P*-value <0.05. Of those genes, we retained cases where there was at least one copy number difference in population mean in at least one pairwise comparison. The resulting 227 genes were analyzed using the post hoc Tukey's HSD test to determine which means were different.

### Ethics statement

The animals used in this study belong to nonprotected species. Permits for catching the mice were not required at the time that they were caught. Some specimens were caught on the properties of private landowners, with oral permission from the landowners to enter the property and catch mice. The mice were trapped in live traps and provided with food and shelter by experienced personnel under the direction of D.T. Trapping was only conducted in moderate temperature conditions to ensure that there was no danger that the trapped animals would suffer from heat or cold. After trapping, the mice were transferred into standard mouse cages providing food, water, and shelter. Transportation to the laboratory, maintenance, and handling were conducted in accordance with German animal welfare law (Tierschutzgesetz) and FELASA guidelines. Permits for keeping mice were obtained from the local veterinary office “Veterinäramt Kreis Plön” (permit number: 1401-144/PLÖ-004697).

## Data access

All CNVs were deposited into the database of genomic structural variation (dbVAR; http://www.ncbi.nlm.nih.gov/dbvar/) under accession number nstd95. The raw sequence reads were deposited in the European Nucleotide Archive (ENA; http://www.ebi.ac.uk/ena/) under project accession number PRJEB9450.

## Supplementary Material

Supplemental Material

## References

[PEZERGR187187C1] Abyzov A, Urban AE, Snyder M, Gerstein M. 2011 CNVnator: an approach to discover, genotype, and characterize typical and atypical CNVs from family and population genome sequencing. Genome Res 21: 974–984. 21324876 10.1101/gr.114876.110 PMC3106330

[PEZERGR187187C2] Alexandrov A, Colognori D, Shu M, Steitz JA. 2012 Human spliceosomal protein CWC22 plays a role in coupling splicing to exon junction complex deposition and nonsense-mediated decay. Proc Natl Acad Sci 109: 21313–21318. 23236153 10.1073/pnas.1219725110 PMC3535618

[PEZERGR187187C3] Alkan C, Coe BP, Eichler EE. 2011 Genome structural variation discovery and genotyping. Nat Rev Genet 12: 363–376. 21358748 10.1038/nrg2958 PMC4108431

[PEZERGR187187C4] Axelsson E, Ratnakumar A, Arendt M-L, Maqbool K, Webster MT, Perloski M, Liberg O, Arnemo JM, Hedhammar A, Lindblad-Toh K. 2013 The genomic signature of dog domestication reveals adaptation to a starch-rich diet. Nature 495: 360–364. 23354050 10.1038/nature11837

[PEZERGR187187C5] Börsch-Haubold AG, Montero I, Konrad K, Haubold B. 2014 Genome-wide quantitative analysis of histone H3 lysine 4 trimethylation in wild house mouse liver: environmental change causes epigenetic plasticity. PLoS One 9: e97568. 24849289 10.1371/journal.pone.0097568 PMC4029994

[PEZERGR187187C6] Bryk J, Tautz D. 2014 Copy number variants and selective sweeps in natural populations of the house mouse (*Mus musculus domesticus*). Front Genet 5: 153. 24917877 10.3389/fgene.2014.00153 PMC4042557

[PEZERGR187187C7] Chain FJJ, Feulner PGD, Panchal M, Eizaguirre C, Samonte IE, Kalbe M, Lenz TL, Stoll M, Bornberg-Bauer E, Milinski M, 2014 Extensive copy-number variation of young genes across stickleback populations. PLoS Genet 10: e1004830. 25474574 10.1371/journal.pgen.1004830 PMC4256280

[PEZERGR187187C8] Chamero P, Marton TF, Logan DW, Flanagan K, Cruz JR, Saghatelian A, Cravatt BF, Stowers L. 2007 Identification of protein pheromones that promote aggressive behavior. Nature 450: 899–902. 18064011 10.1038/nature05997

[PEZERGR187187C9] Chen WK, Swartz JD, Rush LJ, Alvarez CE. 2009 Mapping DNA structural variation in dogs. Genome Res 19: 500–509. 19015322 10.1101/gr.083741.108 PMC2661804

[PEZERGR187187C10] Chen C, Qiao R, Wei R, Guo Y, Ai H, Ma J, Ren J, Huang L. 2012 A comprehensive survey of copy number variation in 18 diverse pig populations and identification of candidate copy number variable genes associated with complex traits. BMC Genomics 13: 733. 23270433 10.1186/1471-2164-13-733 PMC3543711

[PEZERGR187187C11] Conrad DF, Andrews TD, Carter NP, Hurles ME, Pritchard JK. 2006 A high-resolution survey of deletion polymorphism in the human genome. Nat Genet 38: 75–81. 16327808 10.1038/ng1697

[PEZERGR187187C12] Cooper GM, Nickerson DA, Eichler EE. 2007 Mutational and selective effects on copy-number variants in the human genome. Nat Genet 39: S22–S29. 17597777 10.1038/ng2054

[PEZERGR187187C13] Cucchi T, Vigne JD, Auffray JC. 2005 First occurrence of the house mouse (*Mus musculus domesticus* Schwarz & Schwarz, 1943) in the Western Mediterranean: a zooarchaeological revision of subfossil occurrences. Biol J Linn Soc 84: 429–445.

[PEZERGR187187C14] Cutler G, Marshall LA, Chin N, Baribault H, Kassner PD. 2007 Significant gene content variation characterizes the genomes of inbred mouse strains. Genome Res 17: 1743–1754. 17989247 10.1101/gr.6754607 PMC2099583

[PEZERGR187187C15] Didion JP, Morgan AP, Clayshulte AM, Mcmullan RC, Yadgary L, Petkov PM, Bell TA, Gatti DM, Crowley JJ, Hua K, 2015 A multi-megabase copy number gain causes maternal transmission ratio distortion on mouse chromosome 2. PLoS Genet 11: e1004850. 25679959 10.1371/journal.pgen.1004850 PMC4334553

[PEZERGR187187C16] Doan R, Cohen N, Harrington J, Veazey K, Juras R, Cothran G, McCue ME, Skow L, Dindot SV. 2012 Identification of copy number variants in horses. Genome Res 22: 899–907. 22383489 10.1101/gr.128991.111 PMC3337435

[PEZERGR187187C17] Duan J, Zhang JG, Deng HW, Wang YP. 2013 Comparative studies of copy number variation detection methods for next-generation sequencing technologies. PLoS One 8: e59128. 23527109 10.1371/journal.pone.0059128 PMC3604020

[PEZERGR187187C18] Eden E, Navon R, Steinfeld I, Lipson D, Yakhini Z. 2009 Gorilla: a tool for discovery and visualization of enriched GO terms in ranked gene lists. BMC Bioinformatics 10: 48. 19192299 10.1186/1471-2105-10-48 PMC2644678

[PEZERGR187187C19] Egan CM, Sridhar S, Wigler M, Hall IM. 2007 Recurrent DNA copy number variation in the laboratory mouse. Nat Genet 39: 1384–1389. 17965714 10.1038/ng.2007.19

[PEZERGR187187C20] Eichler EE. 2001 Recent duplication, domain accretion and the dynamic mutation of the human genome. Trends Genet 17: 661–669. 11672867 10.1016/s0168-9525(01)02492-1

[PEZERGR187187C21] Ewing AD, Ballinger TJ, Earl D; Broad Institute Genome Sequencing and Analysis Program and Platform, Harris CC, Ding L, Wilson RK, Haussler D. 2013 Retrotransposition of gene transcripts leads to structural variation in mammalian genomes. Genome Biol 14: R22. 23497673 10.1186/gb-2013-14-3-r22 PMC3663115

[PEZERGR187187C22] Feuk L, Marshall CR, Wintle RF, Scherer SW. 2006 Structural variants: changing the landscape of chromosomes and design of disease studies. Hum Mol Genet 15: R57–R66. 16651370 10.1093/hmg/ddl057

[PEZERGR187187C23] Gazave E, Darre F, Morcillo-Suarez C, Petit-Marty N, Carreno A, Marigorta UM, Ryder OA, Blancher A, Rocchi M, Bosch E. 2011 Copy number variation analysis in the great apes reveals species-specific patterns of structural variation. Genome Res 21: 1626–1639. 21824994 10.1101/gr.117242.110 PMC3202280

[PEZERGR187187C24] Girirajan S, Campbell CD, Eichler EE. 2011 Human copy number variation and complex genetic disease. Annu Rev Genet 45: 203–226. 21854229 10.1146/annurev-genet-102209-163544 PMC6662611

[PEZERGR187187C25] Gonzalez E, Kulkarni H, Bolivar H, Mangano A, Sanchez R, Catano G, Nibbs RJ, Freedman BI, Quinones MP, Bamshad MJ, 2005 The influence of *CCL3L1* gene–containing segmental duplications on HIV-1/AIDS susceptibility. Science 307: 1434–1440. 15637236 10.1126/science.1101160

[PEZERGR187187C26] Graubert TA, Cahan P, Edwin D, Selzer RR, Richmond TA, Eis PS, Shannon WD, Li X, McLeod HL, Cheverud JM, 2007 A high-resolution map of segmental DNA copy number variation in the mouse genome. PLoS Genet 3: e3. 17206864 10.1371/journal.pgen.0030003 PMC1761046

[PEZERGR187187C27] Guénet JL, Bonhomme F. 2003 Wild mice: an ever-increasing contribution to a popular mammalian model. Trends Genet 19: 24–31. 12493245 10.1016/s0168-9525(02)00007-0

[PEZERGR187187C28] Guryev V, Saar K, Adamovic T, Verheul M, van Heesch SA, Cook S, Pravenec M, Aitman T, Jacob H, Shull JD, 2008 Distribution and functional impact of DNA copy number variation in the rat. Nat Genet 40: 538–545. 18443591 10.1038/ng.141

[PEZERGR187187C29] Hardouin EA, Orth A, Teschke M, Darvish J, Tautz D, Bonhomme F. 2015 Eurasian house mouse (*Mus musculus* L.) differentiation at microsatellite loci identifies the Iranian plateau as a phylogeographic hotspot. BMC Evol Biol 15: 26. 25888407 10.1186/s12862-015-0306-4 PMC4342898

[PEZERGR187187C30] Henrichsen CN, Chaignat E, Reymond A. 2009a Copy number variants, diseases and gene expression. Hum Mol Genet 18: R1–R8. 19297395 10.1093/hmg/ddp011

[PEZERGR187187C31] Henrichsen CN, Vinckenbosch N, Zöllner S, Chaignat E, Pradervand S, Schütz F, Ruedi M, Kaessmann H, Reymond A. 2009b Segmental copy number variation shapes tissue transcriptomes. Nat Genet 41: 424–429. 19270705 10.1038/ng.345

[PEZERGR187187C32] Heo JI, Cho JH, Kim JR. 2013 HJURP regulates cellular senescence in human fibroblasts and endothelial cells via a p53-dependent pathway. J Gerontol A Biol Sci Med Sci 68: 914–925. 23292286 10.1093/gerona/gls257

[PEZERGR187187C33] Hirase S, Ozaki H, Iwasaki W. 2014 Parallel selection on gene copy number variations through evolution of three-spined stickleback genomes. BMC Genomics 15: 735. 25168270 10.1186/1471-2164-15-735 PMC4159527

[PEZERGR187187C34] Hurst JL, Payne CE, Nevison CM, Marie AD, Humphries RE, Robertson DH, Cavaggioni A, Beynon RJ. 2001 Individual recognition in mice mediated by major urinary proteins. Nature 414: 631–634. 11740558 10.1038/414631a

[PEZERGR187187C35] Ihle S, Ravaoarimanana I, Thomas M, Tautz D. 2006 An analysis of signatures of selective sweeps in natural populations of the house mouse. Mol Biol Evol 23: 790–797. 16421176 10.1093/molbev/msj096

[PEZERGR187187C36] Iskow RC, Gokcumen O, Lee C. 2012 Exploring the role of copy number variants in human adaptation. Trends Genet 28: 245–257. 22483647 10.1016/j.tig.2012.03.002 PMC3533238

[PEZERGR187187C37] Itsara A, Cooper GM, Baker C, Girirajan S, Li J, Absher D, Krauss RM, Myers RM, Ridker PM, Chasman DI, 2009 Population analysis of large copy number variants and hotspots of human genetic disease. Am J Hum Genet 84: 148–161. 19166990 10.1016/j.ajhg.2008.12.014 PMC2668011

[PEZERGR187187C38] Jakobsson M, Scholz SW, Scheet P, Gibbs JR, VanLiere JM, Fung HC, Szpiech ZA, Degnan JH, Wang K, Guerreiro R, 2008 Genotype, haplotype and copy-number variation in worldwide human populations. Nature 451: 998–1003. 18288195 10.1038/nature06742

[PEZERGR187187C39] Katju V, Bergthorsson U. 2014 Copy-number changes in evolution: rates, fitness effects and adaptive significance. Front Genet 4: 273. 24368910 10.3389/fgene.2013.00273 PMC3857721

[PEZERGR187187C40] Keane TM, Goodstadt L, Danecek P, White MA, Wong K, Yalcin B, Heger A, Agam A, Slater G, Goodson M, 2011 Mouse genomic variation and its effect on phenotypes and gene regulation. Nature 477: 289–294. 21921910 10.1038/nature10413 PMC3276836

[PEZERGR187187C41] Keane TM, Wong K, Adams DJ, Flint J, Reymond A, Yalcin B. 2014 Identification of structural variation in mouse genomes. Front Genet 5: 192. 25071822 10.3389/fgene.2014.00192 PMC4079067

[PEZERGR187187C42] Kilmartin JV. 2003 Sfi1p has conserved centrin-binding sites and an essential function in budding yeast spindle pole body duplication. J Cell Biol 162: 1211–1221. 14504268 10.1083/jcb.200307064 PMC2173958

[PEZERGR187187C43] Kimura M. 1983 The neutral theory of molecular evolution. Cambridge University Press, Cambridge.

[PEZERGR187187C44] Krzywinski M, Schein J, Birol I, Connors J, Gascoyne R, Horsman D, Jones SJ, Marra MA. 2009 Circos: an information aesthetic for comparative genomics. Genome Res 19: 1639–1645. 19541911 10.1101/gr.092759.109 PMC2752132

[PEZERGR187187C45] Li H, Durbin R. 2009 Fast and accurate short read alignment with Burrows–Wheeler Transform. Bioinformatics 25: 1754–1760. 19451168 10.1093/bioinformatics/btp324 PMC2705234

[PEZERGR187187C46] Li H, Handsaker B, Wysoker A, Fennell T, Ruan J, Homer N, Marth G, Abecasis G, Durbin R. 2009 The Sequence Alignment/Map format and SAMtools. Bioinformatics 25: 2078–2079. 19505943 10.1093/bioinformatics/btp352 PMC2723002

[PEZERGR187187C47] Liu EY, Morgan AP, Chesler EJ, Wang W, Churchill GA, de Villena FP. 2014 High-resolution sex-specific linkage maps of the mouse reveal polarized distribution of crossovers in male germline. Genetics 197: 91–106. 24578350 10.1534/genetics.114.161653 PMC4012503

[PEZERGR187187C48] Logan DW, Marton TF, Stowers L. 2008 Species specificity in major urinary proteins by parallel evolution. PLoS One 3: e3280. 18815613 10.1371/journal.pone.0003280 PMC2533699

[PEZERGR187187C49] Marchlewska-Koj A, Cavaggioni A, Mucignat-Caretta C, Olejniczak P. 2000 Stimulation of estrus in female mice by male urinary proteins. J Chem Ecol 26: 2355–2365.

[PEZERGR187187C50] McCarroll SA, Hadnott TN, Perry GH, Sabeti PC, Zody MC, Barrett JC, Dallaire S, Gabriel SB, Lee C, Daly MJ, 2006 Common deletion polymorphisms in the human genome. Nat Genet 38: 86–92. 16468122 10.1038/ng1696

[PEZERGR187187C51] Mills RE, Walter K, Stewart C, Handsaker RE, Chen K, Alkan C, Abyzov A, Yoon SC, Ye K, Cheetham RK, 2011 Mapping copy number variation by population-scale genome sequencing. Nature 470: 59–65. 21293372 10.1038/nature09708 PMC3077050

[PEZERGR187187C52] Mishra PK, Au WC, Choy JS, Kuich PH, Baker RE, Foltz DR, Basrai MA. 2011 Misregulation of Scm3p/HJURP causes chromosome instability in *Saccharomyces cerevisiae* and human cells. PLoS Genet 7: e1002303. 21980305 10.1371/journal.pgen.1002303 PMC3183075

[PEZERGR187187C53] Montero I, Teschke M, Tautz D. 2013 Paternal imprinting of mating preferences between natural populations of house mice (*Mus musculus domesticus*). Mol Ecol 22: 2549–2562. 23506395 10.1111/mec.12271

[PEZERGR187187C54] More L. 2006 Mouse major urinary proteins trigger ovulation via the vomeronasal organ. Chem Senses 31: 393–401. 16510842 10.1093/chemse/bjj043

[PEZERGR187187C55] Neme R, Tautz D. 2014 Evolution: dynamics of *de novo* gene emergence. Curr Biol 24: R238–R240. 24650912 10.1016/j.cub.2014.02.016

[PEZERGR187187C56] Nguyen D-Q, Webber C, Hehir-Kwa J, Pfundt R, Veltman J, Ponting CP. 2008 Reduced purifying selection prevails over positive selection in human copy number variant evolution. Genome Res 18: 1711–1723. 18687881 10.1101/gr.077289.108 PMC2577867

[PEZERGR187187C57] Orozco LD, Cokus SJ, Ghazalpour A, Ingram-Drake L, Wang S, van Nas A, Che N, Araujo JA, Pellegrini M, Lusis AJ. 2009 Copy number variation influences gene expression and metabolic traits in mice. Hum Mol Genet 18: 4118–4129. 19648292 10.1093/hmg/ddp360 PMC2758141

[PEZERGR187187C58] Pei B, Sisu C, Frankish A, Howald C, Habegger L, Mu X, Harte R, Balasubramanian S, Tanzer A, Diekhans M, 2012 The GENCODE pseudogene resource. Genome Biol 13: R51. 22951037 10.1186/gb-2012-13-9-r51 PMC3491395

[PEZERGR187187C59] Perry GH, Tchinda J, McGrath SD, Zhang J, Picker SR, Caceres AM, Iafrate AJ, Tyler-Smith C, Scherer SW, Eichler EE. 2006 Hotspots for copy number variation in chimpanzees and humans. Proc Natl Acad Sci 103: 8006–8011. 16702545 10.1073/pnas.0602318103 PMC1472420

[PEZERGR187187C60] Perry GH, Dominy NJ, Claw KG, Lee AS, Fiegler H, Redon R, Werner J, Villanea FA, Mountain JL, Misra R, 2007 Diet and the evolution of human amylase gene copy number variation. Nat Genet 39: 1256–1260. 17828263 10.1038/ng2123 PMC2377015

[PEZERGR187187C61] Quinlan AR, Hall IM. 2010 BEDTools: a flexible suite of utilities for comparing genomic features. Bioinformatics 26: 841–842. 20110278 10.1093/bioinformatics/btq033 PMC2832824

[PEZERGR187187C62] Quinlan AR, Clark RA, Sokolova S, Leibowitz ML, Zhang Y, Hurles ME, Mell JC, Hall IM. 2010 Genome-wide mapping and assembly of structural variant breakpoints in the mouse genome. Genome Res 20: 623–635. 20308636 10.1101/gr.102970.109 PMC2860164

[PEZERGR187187C63] Rajabi-Maham H, Orth A, Bonhomme F. 2008 Phylogeography and postglacial expansion of *Mus musculus domesticus* inferred from mitochondrial DNA coalescent, from Iran to Europe. Mol Ecol 17: 627–641. 18179435 10.1111/j.1365-294X.2007.03601.x

[PEZERGR187187C64] Raychaudhuri S, Korn JM, McCarroll SA; International Schizophrenia Consortium, Altshuler D, Sklar P, Purcell S, Daly MJ. 2010 Accurately assessing the risk of schizophrenia conferred by rare copy-number variation affecting genes with brain function. PLoS Genet 6: e1001097. 20838587 10.1371/journal.pgen.1001097 PMC2936523

[PEZERGR187187C65] Redon R, Ishikawa S, Fitch KR, Feuk L, Perry GH, Andrews TD, Fiegler H, Shapero MH, Carson AR, Chen W, 2006 Global variation in copy number in the human genome. Nature 444: 444–454. 17122850 10.1038/nature05329 PMC2669898

[PEZERGR187187C66] Reichstein H, Vauk G. 1967 Beitrag zur Kenntnis der Helgolander Hausmaus, *Mus musculus helgolandicus* Zimmermann, 1953. Verh Dtsch Zool Ges 31: 386–394.

[PEZERGR187187C67] Scherer SW, Lee C, Birney E, Altshuler DM, Eichler EE, Carter NP, Hurles ME, Feuk L. 2007 Challenges and standards in integrating surveys of structural variation. Nat Genet 39: S7–S15. 17597783 10.1038/ng2093 PMC2698291

[PEZERGR187187C68] Schlötterer C, Tautz D. 1994 Chromosomal homogeneity of *Drosophila* ribosomal DNA arrays suggests intrachromosomal exchanges drive concerted evolution. Curr Biol 4: 777–783. 7820547 10.1016/s0960-9822(00)00175-5

[PEZERGR187187C69] Schrider DR, Hahn MW. 2010 Gene copy-number polymorphism in nature. Proc R Soc Lond B Biol Sci 277: 3213–3221. 10.1098/rspb.2010.1180 PMC2981937 20591863

[PEZERGR187187C70] Schrider DR, Navarro FC, Galante PA, Parmigiani RB, Camargo AA, Hahn MW, de Souza SJ. 2013 Gene copy-number polymorphism caused by retrotransposition in humans. PLoS Genet 9: e1003242. 23359205 10.1371/journal.pgen.1003242 PMC3554589

[PEZERGR187187C71] Sebat J, Lakshmi B, Troge J, Alexander J, Young J, Lundin P, Månér S, Massa H, Walker M, Chi M, 2004 Large-scale copy number polymorphism in the human genome. Science 305: 525–528. 15273396 10.1126/science.1098918

[PEZERGR187187C72] Sharp AJ, Locke DP, McGrath SD, Cheng Z, Bailey JA, Vallente RU, Pertz LM, Clark RA, Schwartz S, Segraves R, 2005 Segmental duplications and copy-number variation in the human genome. Am J Hum Genet 77: 78–88. 15918152 10.1086/431652 PMC1226196

[PEZERGR187187C73] She X, Cheng Z, Zöllner S, Church DM, Eichler EE. 2008 Mouse segmental duplication and copy number variation. Nat Genet 40: 909–914. 18500340 10.1038/ng.172 PMC2574762

[PEZERGR187187C74] Shlien A, Tabori U, Marshall CR, Pienkowska M, Feuk L, Novokmet A, Nanda S, Druker H, Scherer SW, Malkin D. 2008 Excessive genomic DNA copy number variation in the Li–Fraumeni cancer predisposition syndrome. Proc Natl Acad Sci 105: 11264–11269. 18685109 10.1073/pnas.0802970105 PMC2516272

[PEZERGR187187C75] Stankiewicz P, Lupski JR. 2002 Genome architecture, rearrangements and genomic disorders. Trends Genet 18: 74–82. 11818139 10.1016/s0168-9525(02)02592-1

[PEZERGR187187C76] Stankiewicz P, Lupski JR. 2010 Structural variation in the human genome and its role in disease. Annu Rev Med 61: 437–455. 20059347 10.1146/annurev-med-100708-204735

[PEZERGR187187C77] Staubach F, Lorenc A, Messer PW, Tang K, Petrov DA, Tautz D. 2012 Genome patterns of selection and introgression of haplotypes in natural populations of the house mouse (*Mus musculus*). PLoS Genet 8: e1002891. 22956910 10.1371/journal.pgen.1002891 PMC3431316

[PEZERGR187187C78] Steckelberg AL, Boehm V, Gromadzka AM, Gehring NH. 2012 CWC22 connects pre-mRNA splicing and exon junction complex assembly. Cell Rep 2: 454–461. 22959432 10.1016/j.celrep.2012.08.017

[PEZERGR187187C79] Stellfox ME, Bailey AO, Foltz DR. 2013 Putting CENP-A in its place. Cell Mol Life Sci 70: 387–406. 22729156 10.1007/s00018-012-1048-8 PMC4084702

[PEZERGR187187C80] Stingele S, Stoehr G, Peplowska K, Cox J, Mann M, Storchova Z. 2012 Global analysis of genome, transcriptome and proteome reveals the response to aneuploidy in human cells. Mol Syst Biol 8: 608. 22968442 10.1038/msb.2012.40 PMC3472693

[PEZERGR187187C81] Teschke M, Mukabayire O, Wiehe T, Tautz D. 2008 Identification of selective sweeps in closely related populations of the house mouse based on microsatellite scans. Genetics 180: 1537–1545. 18791245 10.1534/genetics.108.090811 PMC2581955

[PEZERGR187187C82] Watkins-Chow DE, Pavan WJ. 2008 Genomic copy number and expression variation within the C57BL/6J inbred mouse strain. Genome Res 18: 60–66. 18032724 10.1101/gr.6927808 PMC2134784

[PEZERGR187187C83] Wong KK, deLeeuw RJ, Dosanjh NS, Kimm LR, Cheng Z, Horsman DE, MacAulay C, Ng RT, Brown CJ, Eichler EE, 2007 A comprehensive analysis of common copy-number variations in the human genome. Am J Hum Genet 80: 91–104. 17160897 10.1086/510560 PMC1785303

[PEZERGR187187C84] Wong K, Bumpstead S, van der Weyden L, Reinholdt LG, Wilming LG, Adams DJ, Keane TM. 2012 Sequencing and characterization of the FVB/NJ mouse genome. Genome Biol 13: R72. 22916792 10.1186/gb-2012-13-8-r72 PMC3491372

[PEZERGR187187C85] Zimmermann K. 1949 Die Hausmaus von Helgoland *Mus musculus helgolandicus* sspec. nov. Z Säugetierkd 17: 163–166.

