# Supplementary material for: Divergence patterns of genic copy number variation in natural populations of the house mouse (Mus musculus domesticus) reveal three conserved genes with major population-specific expansions
Source: Genome Res. 2015 Aug;25(8):1114–24. doi: 10.1101/gr.187187.114 (PMC4509996; doi:10.1101/gr.187187.114)
Supplement: Supplemental Material [file supp_25_8_1114__index.html]

Divergence patterns of genic copy number variation in natural populations of the house mouse (Mus musculus domesticus) reveal three conserved genes with major population-specific expansions — Divergence patterns of genic copy number variation in natural populations of the house mouse (Mus musculus domesticus) reveal three conserved genes with major population-specific expansions — Supplemental Material 

# Divergence patterns of genic copy number variation in natural populations of the house mouse (*Mus musculus domesticus*) reveal three conserved genes with major population-specific expansions

## Supplemental Material

**Files in this Data Supplement:**

- Supplemental Material.pdf
- Supp Table S4.xlsx
- Supp Table S5.xlsx
- Supp Table S7.xlsx
- Supp Table S8.xlsx
