# Supplementary material for: Divergence patterns of genic copy number variation in natural populations of the house mouse (Mus musculus domesticus) reveal three conserved genes with major population-specific expansions
Source: Genome Res. 2015 Aug;25(8):1114–24. doi: 10.1101/gr.187187.114 (PMC4509996; doi:10.1101/gr.187187.114)
Supplement: Supplemental Material [file supp_gr.187187.114_Supplemental_Material.pdf]

Divergence patterns of genic copy number variation in natural populations of the house mouse (*Mus musculus domesticus*) reveal three conserved genes with major population-specific expansions

Željka Pezer, Bettina Harr, Meike Teschke, Hiba Babiker and Diethard Tautz

Supplemental Material

Supplemental Text

Text S1: Reasoning for using a read-depth approach for CNV detection 3
Text S2: Digital PCR validation and estimate of false discovery rate 5
Text S3: Reduced CNV detection power due to lower read coverage 8
Text S4: Control for false positive singleton CNVs 9
Text S5: Comparisons between populations and with inbred mouse strains 10
Text S6: Assessment of CNV detection in regions of high similarity 13
Text S7: CNVR size *vs.* frequency 14
Text S8: Size of neural genes and its influence on enrichment at CNVRs 16
Text S9: Assessment of genotyping accuracy 17
Text S10: Validation of deleted genes analysis 17
Text S11: Validation of V_{ST} analysis 19
Text S12: Outlier analysis 19

Supplemental Figures

Figure S1: Definition of CNV classes used throughout the analysis 4
Figure S2: Validation of CNV loci by droplet digital PCR 6
Figure S3: CNV detection power *vs.* read coverage 8
Figure S4: Detected singleton CNVs at varying cutoffs for normalized RD signal 9
Figure S5: Pairwise similarity matrix for wild mouse individuals 10
Figure S6: Comparison with CNVs from inbred mouse strains 12
Figure S7: Assessment of CNV detection in regions of high similarity 14
Figure S8: Distribution of CNVR size *vs.* frequency 15
Figure S9: CNVRs intersecting with genes and large segmental duplications 15
Figure S10: Distribution of neural genes to other genes 16
Figure S11: Genotyping accuracy of CNV genes 17
Figure S12: Validation of deleted genes analysis 17
Figure S13: Read depth at CNVs encompassing *Cwc22, Hjurp* and *Sfi1* 18
Figure S14: Validation of V_{ST} analysis 19
Figure S15: Differences in population average gene copy number 20
Figure S16: UCSC Genome Browser view of CNV in the *Mup* locus 21

Supplemental Tables

Table S1. Read mapping and CNV discovery statistics 22
Table S2: GO enrichment analysis of genes in CNVRs overlapping SDs > 10 kb 23
Table S3: GO enrichment analysis of genes in CNVRs not overlapping SDs > 10 kb 24

Table S4: Estimated gene copy number per individual and CNV gene - **separate Excel file**
Table S5: GO term enrichment analysis of CNV genes 25
Table S6. Frequency of deletion alleles - **separate Excel file**
Table S7: Vst values of CNV genes - **separate Excel file**
Table S8. Genes with significant difference in variance of copy number between populations - **separate Excel file**
Table S9: Diploid copy number of genes in amylase cluster by individual 26
Table S10: Diploid copy number of genes in *Mup* cluster by individual 27
Table S11: Pseudogene-parent gene pairs used for copy number correlation analysis 28
Table S12: Assays used for ddPCR 29

References **30**

Text S1: Reasoning for using a read-depth approach for CNV detection

Genome-wide studies of structural variations initially employed microarray techniques but these are now rapidly becoming replaced with more powerful next-generation sequencing (NGS) platforms. Some of the advantages of NGS based approaches include much better genome coverage and resolution, more accurate copy number estimate, and capability to detect novel variants (reviewed in Zhao et al. 2013). NGS based structural variation detection methods rely on short read placement and can be classified into several different strategies, based on the use of mapping information. Each approach has certain advantages and limitations and the choice of method should include careful consideration of potential technical artifacts and how they affect data interpretation. For example, tools based on discordantly mapped paired reads (paired-end mapping; PEM) have high sensitivity and can detect balanced rearrangements such as inversions and translocations, but are at the same time strongly biased towards detection of structural variants smaller than 1 kb. Furthermore, because a sequenced library contains a distribution of insert sizes rather than one discrete value, and because PEM strategy uses a fixed cutoff at which insert length is considered to be anomalous, high false positive and/or negative discovery rates can be expected (Medvedev et al. 2009). On the other hand, read depth (RD) methods are based on a concept that the depth of coverage of a genomic region positively correlates with the copy number of that region and usually employ statistical models to deal with mapping biases and variations (Zhao et al. 2013). Unlike PEM based strategy, RD analysis is not applicable for finding copy number neutral rearrangements and novel insertions that are not already present in the reference genome. However, RD based tools detect large events with maximum sensitivity even at low coverage, and the reliability of a call actually increases with the size of the event (Medvedev et al. 2009; Abyzov et al. 2011; Zhao et al. 2013). Moreover, the RD approach can accurately predict copy numbers and thus perform genotyping, a feature that is beyond those of other NGS based methods. Another advantage of the RD based strategy is the ability to detect variation within repetitive regions of the genome by considering read placement at multiple positions, whereas other NGS based methods are restricted to unique genomic regions (Magi et al. 2012). The power to detect structural variants in low-complexity regions such as SDs is of indisputable value, given that these regions show substantial copy number variation (Sebat et al. 2004; Sharp et al 2005; Cooper et al. 2007; Egan et al. 2007; Medvedev et al. 2009).

Having all the above in mind, our choice of methodology was governed by the following objectives:

1) finding large variations which would encompass whole genes;
2) comparison of actual copy numbers in order to infer differentiating patterns;
3) detecting variants in SDs regions which are known to exhibit great deal of polymorphism in population;
4) minimizing possibility of the influence of technical artifacts on data interpretation when comparing between samples of varying sequencing coverage.

To this end, RD approach of CNV detection was chosen as the most appropriate or even the only applicable methodology. Admittedly, a combination of different approaches such as for example RD and PEM would enrich our collection of identified CNVs, however, it could also misguide the data interpretation. For example, lower sequencing coverage is

associated with lower sensitivity, *i.e.* inability to detect much CNVs, especially those of smaller size. Hence, the events readily detected in samples of better coverage could go undetected in samples of lower resolution and misinterpreted as lacking. This could easily lead to erroneous conclusions associated with presence-absence patterns and population differentiation analysis. By focusing on larger events (≥ 1 kb) and by employing RD strategy which is much more robust to coverage differences, we attempt to avoid these issues. Furthermore, for each of the aforementioned objectives, we tackle the possibility of misinterpretation caused by technical issues by performing suitable control analyses (see further in the text).

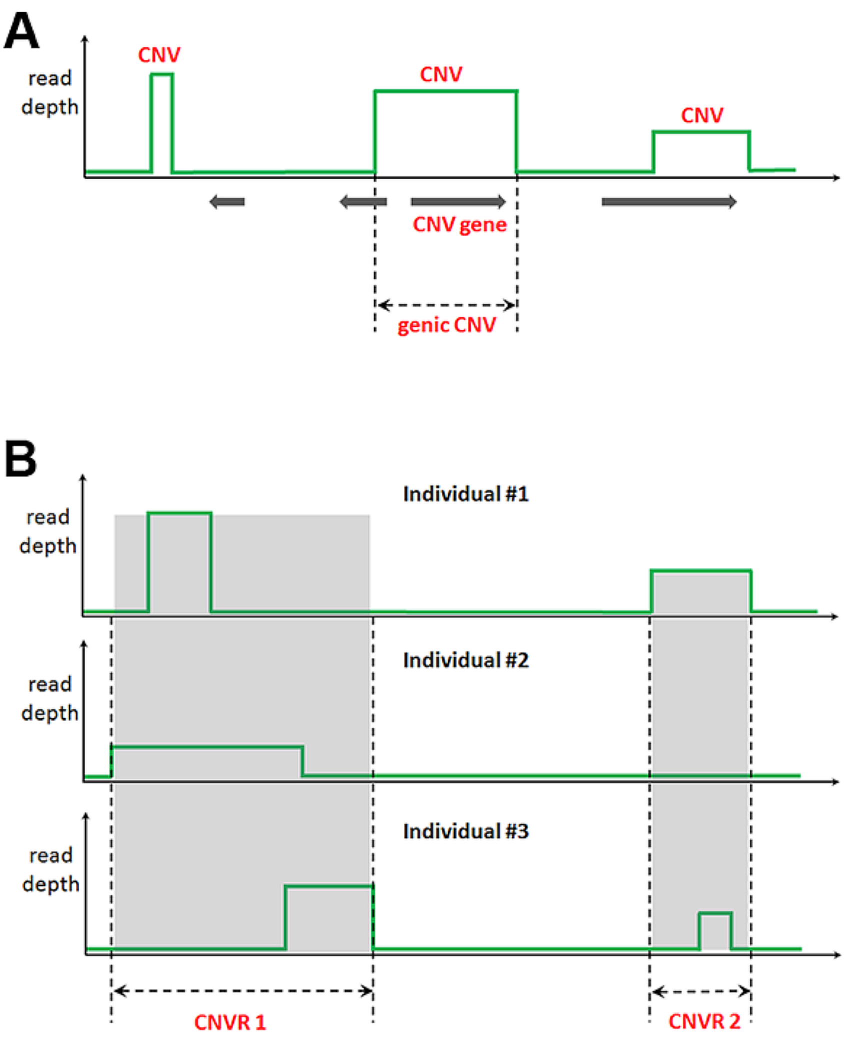

Figure S1. Definition of CNV classes used throughout the analysis. For simplicity, only duplications are depicted as CNVs in this schematic representation as peaks of read depth (green lines); however, both duplications and deletions are considered in the analysis. Arrows represent genes (transcription units). **(A)** "CNV" is any region ≥ 1kb with read depth differences identified by CNVnator; "genic CNV" is a call that contains at least one full gene which, in that case, is called "CNV Gene". **(B)** All overlapping calls across individuals merged together define a Copy Number Variable Region "CNVR".

Text S2: Digital PCR validation and estimate of false discovery rate

For ddPCR validation, we selected CNVs at different genomic regions that either represent various predicted copy number ranges across all 27 individuals or showed population differentiation in our dataset. Of the 44 considered CNVs, we were able to design specific primers and probes for 21 different loci (see suppl. Table S12). This was not surprising, given the association of copy umber polymorphism with repetitive regions which hinders amplification of specific targets. Additionally, for two genes, *Luzp4* and *Gm21671*, we were able to design assays which contained non-specific primers. Amplification of unwanted targets was prevented by additional digestion of DNA with MseI prior to ddPCR, which cuts inside non-specific targets while leaving the desired target intact. The resulting copy number was compared with the CNVnator-determined copy number for the same region in all 27 individuals (Figure S2).

In order to estimate false discovery rate (FDR) of our CNV call set, we used two measures of correlation between the computationally predicted and experimentally determined copy numbers: Pearson's correlation coefficient (*r*) and Lin's Concordance Correlation Coefficient (CCC) (Figure S2). The latter is more stringent in that it measures departure from the equality line (45°) between CNVnator predictions and ddPCR results (Lin 1989). The correlation was considered to be very strong if either *r* or CCC was > 0.7. Those cases where correlation coefficient was calculated to be below 0.7 were considered to be false positives. Based on Pearson's *r*, we find two false positive CNV loci among 23 tested (*Defb8* and *Nxpe5*), resulting in FDR of 8.6%. Based on CCC, we detect five false positives (*Defb8*, *Gm13152-13154*, *Gzma*, *Nxpe5* and *Tex24*), estimating the FDR to be 21.7%.

Figure S2 - Validation of CNV loci by droplet digital PCR

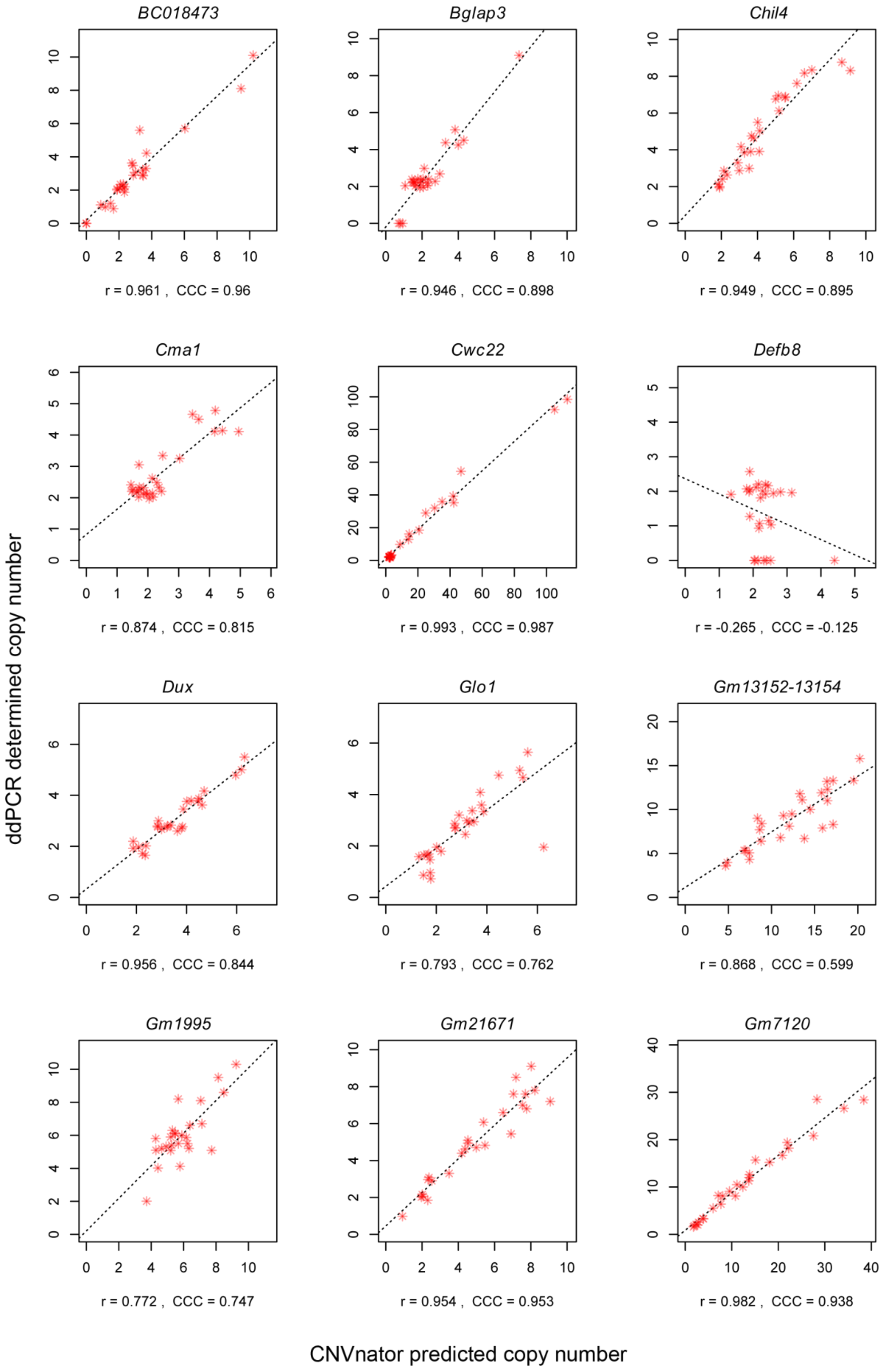

Figure S2 - Continued

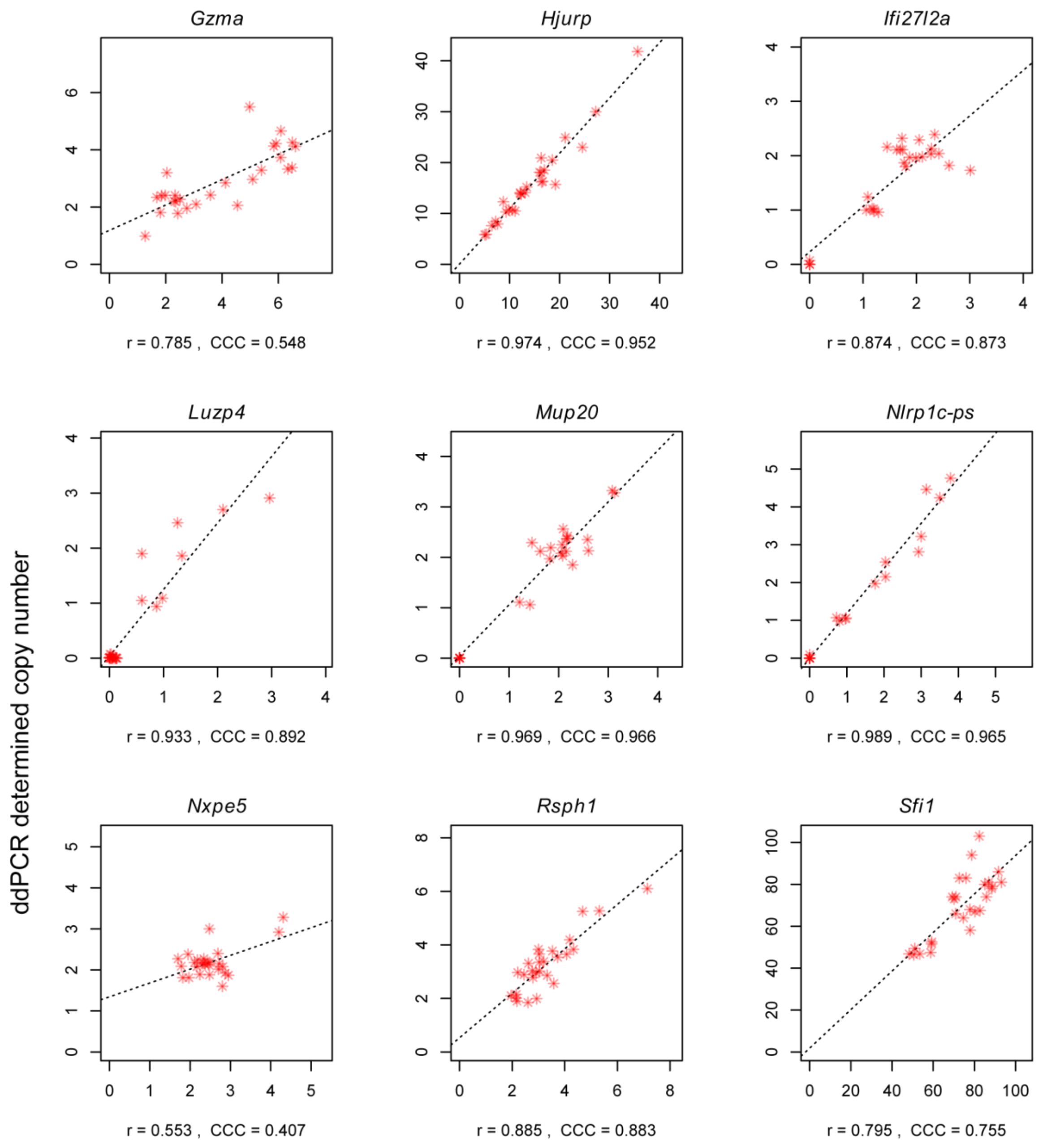

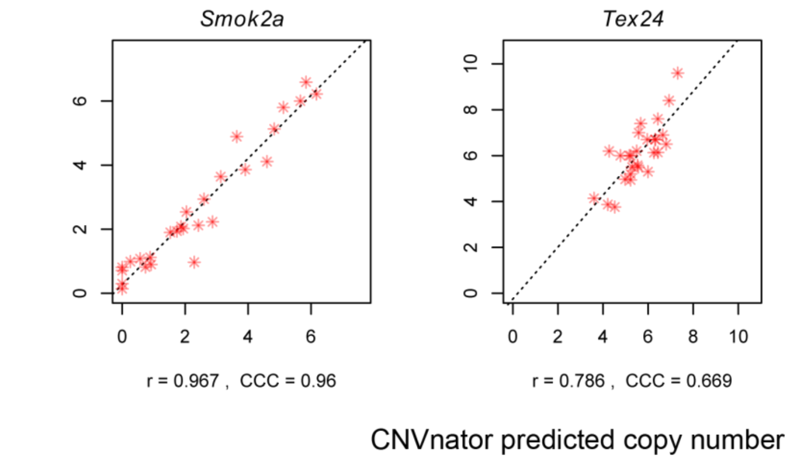

Figure S2. Assays for 23 different loci were designed and used to screen all 27 individuals (red asterisks). The results of ddPCR runs (y axis) were plotted against copy numbers predicted with CNVnator (x axis) for the amplicon regions. Fitted regression lines are shown dashed; *r* is Pearson's correlation coefficient; CCC is Lin's Concordance Correlation Coefficient.

Text S3: Reduced CNV detection power due to lower read coverage

Neighboring CNV calls can appear as one single call in samples with shallower coverage, resulting in a lower proportion of smaller CNVs. Indeed, on average, 79%-81% of calls were 1-10 kb long in the three mainland populations, as opposed to only 69% in HEL (Figure S3A). This effect is also visible from the calculated average CNV length, which for HEL population is substantially larger than for mainland populations (17 kb compared to 11-12 kb, respectively; Table S1 below). When only genic CNVs or CNV genes are considered, which are mostly much larger than 1 kb, the difference of HEL to FRA and GER is also smaller (Figure 1 in main text; Table S1 below).

To assess the effect of read depth on CNV counts, we plotted the number of mapped reads in each sample against the number of detected CNVs. The correlation was strong for all CNV counts (Figure S3B - left; $r = 0.857$, Pearson's coefficient), but weak to insignificant for genic CNVs (Figure S3B - middle; $r = 0.304$) and CNV genes (Figure S3B - right; $r = 0.206$). This indicates that the read depth dependence is of less relevance when assessing CNVs associated with genes. Therefore, we reasoned that by focusing on CNV genes and larger events our analyses should be less influenced by technical artifacts and differences in sequencing depth.

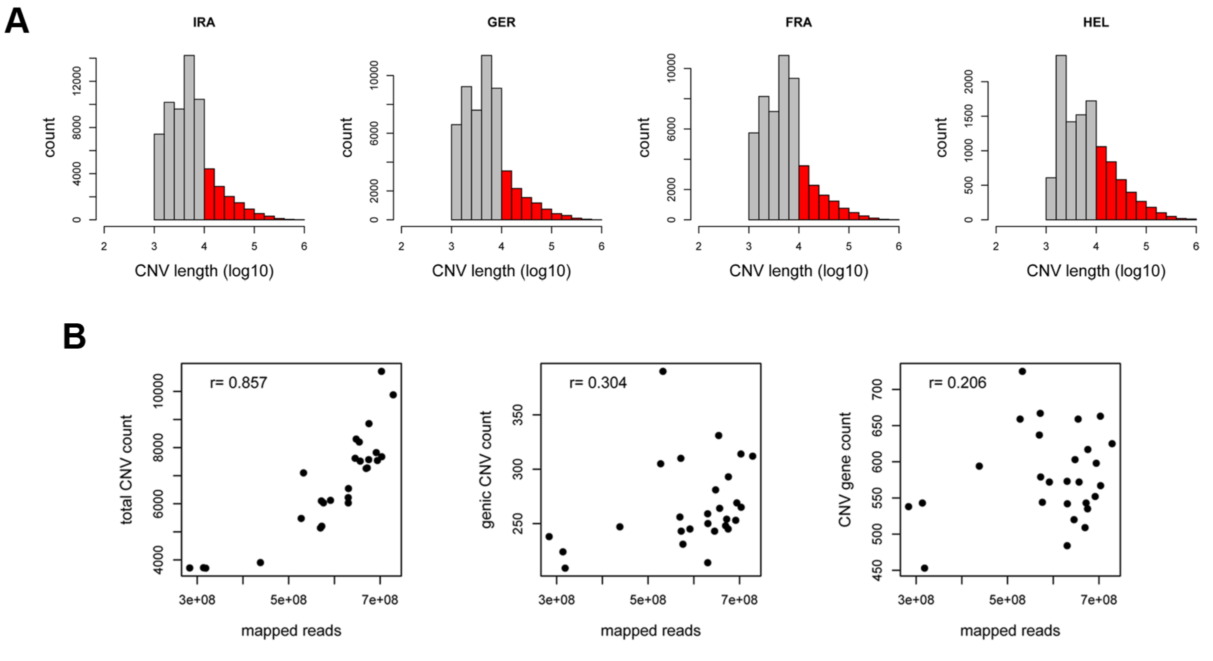

Figure S3. CNV detection power *vs*. read coverage. (A) Difference in CNV size distribution between sample sets. Fraction of calls larger than 10 kb (red) is higher in Heligoland samples compared to mainland samples. This difference reflects weaker resolution of CNV detection as result of lower coverage. **(B)** Correlation between number of mapped reads and CNV counts. *r* is Pearson's coefficient.

Text S4: Control for false positive singleton CNVs

CNVnator calls deletions and duplications when the normalized RD signal is calculated to be below 1.5 (per diploid) and above 2.5, respectively. The more stringent cutoff of 1.4 and 2.6, has been used to find reliable *de novo* CNVs from family trios (Abyzov et al. 2011). Since CNV calling was performed for 27 genomes, to account for multiple comparisons, we tested whether the number of detected singletons would change if the more strict criteria for CNV calling are applied. We varied the normalized RD cutoff, increasingly in its stringency: 1) 1.4 for deletions and 2.6 for duplications; 2) 1.2 and 2.8; and 3) 1.0 and 3.0. For each of the three cutoff sets, we counted the number of detected singletons in each animal and compared their distributions with the original data (Table S1). We detect only borderline significant difference in comparison with the most stringent cutoff applied (Figure S4; $p = 0.0485$ after Bonferroni correction in Dunn's *post hoc* test), indicating that the original data contains *bona fide* singletons in our sample set, rather than false positives.

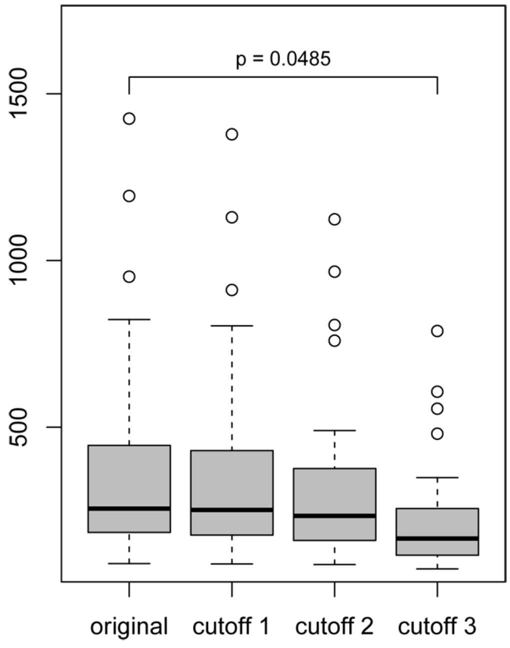

Figure S4. Detected singleton CNVs at varying cutoffs for normalized RD signal applied. Comparison of original dataset with singletons obtained by applying three different cutoffs to call CNVs is shown (see Text S4 for cutoff values). Number of detected singletons for all 27 animals is shown in each box as distribution. Low-significance difference between the original dataset and dataset obtained by applying cutoff 3 was detected and its *p*-adj value is shown (Kruskal-Wallis rank sum test $p = 0.07$).

We detected on average 386 singletons in our sample set (suppl. Table S1), *i.e.* CNV calls found exclusively in a single individual. Considerably more singletons in all IRA mice compared to others is likely due to higher effective population size, consistent with more mutations at low frequency.

We detected on average 180 (median 178, sd = 45) deletions per sample for which the number of mapped reads corresponded to less than 1/100th of a single copy per genome (copy number - CN = 0.01) and can be considered complete deletions of high confidence. In 84 of these on average (81 median, sd = 26) we find no reads aligned at all. If we take CN of 0.5 as the cutoff below which a region is considered absent, over 1% of the genome appears to be deleted compared to the reference assembly. Given the limitations of the read-depth approach, we were not able to identify regions which are present in our samples and absent from the reference genome. However, if a similar outcome applies for vice-versa comparison, the estimated difference in genomic content between the reference assembly and any of our wild mouse samples might exceed 2% of the genome fraction.

Text S5: Comparisons between populations and with inbred mouse strains

Analysis of CNV presence-absence patterns can capture genetic relationships between individuals and popualtions. We defined overlapping CNVs between two individuals as those that intersect by at least 50% of the sequence length (based on position in the reference genome) and counted them in all combinations of comparison between the 27 individuals of the wild populations and for inbred mouse strains. We did not consider calls on the Y chromosome to be able to compare male to female samples.

In the wild mouse samples, two individuals had on average between 2,025-2,759 overlapping CNVs in their respective population (Supplemental Table S1). In each pairwise comparison, the similarity was calculated as the number of overlapping CNVs divided by the average number of detected calls between the two individuals and the resulting similarity matrix is shown in Fig. S5. Based on the number of overlapping CNVs, FRA and GER populations are the most similar to one another. HEL mice share the least number of CNVs with the three other populations, although this could partly be ascribed to the lower number of detected calls. We also observed different degrees of variance in the number of overlapping CNVs within populations. GER mice are more similar to one another than FRA mice, and the IRA population shows the highest diversity.

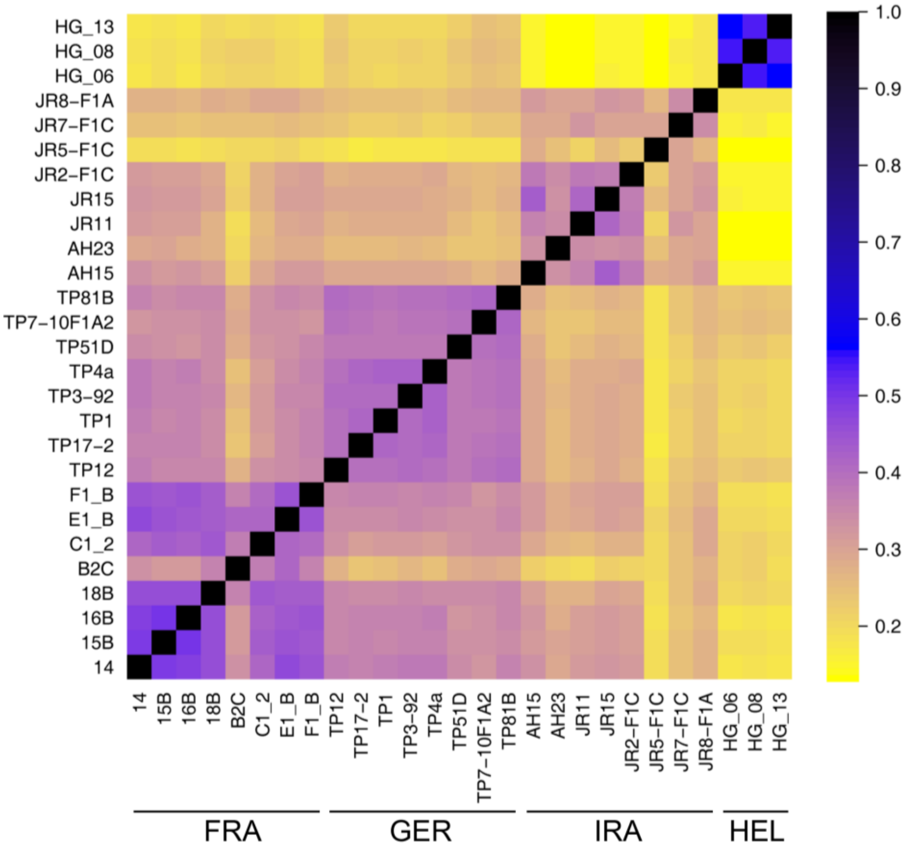

Figure S5: Pairwise similarity matrix based on overlapping CNVs for wild mouse individuals. CNV calls were defined as overlapping between two individuals if their overlap corresponded to a minimum of half of each length (relative to the reference genome). Similarity is presented by a scale from 0 to 1 where value 1 corresponds to absolute identity and 0 means no similarity, *i.e.* no overlapping CNVs. Populations from top to bottom (by rows) are Heligoland (HG samples), Iranian (AH and JR), German (TP) and French (last eight rows).

To estimate the extent to which CNVs from our wild mice samples overlap with those in inbred mice, we downloaded variant calls from ftp://ftp-mouse.sanger.ac.uk/REL-1302-SV/ for 16 mouse strains from the study by Keane et al. (2011; dbVar accession number estd118) and additional strain FVB/NJ from the study by Wong et al. (2012; dbVar accession number estd200). We opted for this release as all calls are relative to the Build 37 (mm9) reference mouse genome C57BL/6J - the same assembly we used for CNV calling. In order to compare it with our set of CNVs, we removed balanced structural variations (SVs) and calls < 1 kb from each of the strains' SV set. Calls on Y chromosomes were not considered to enable comparisons between male and female samples. By using BEDTools (Quinlan and Hall 2010) we intersected calls from each of the 17 inbred strains with calls from each of our 27 wild mice, creating two analysis sets of overlapping calls: those that intersect by at least 1 bp and those that have minimum 50% reciprocal overlap of the reported CNV length with respect to the reference. On average, the inbred strains and wild mice overlapped at 1,679 CNVs (median 1,631) when the minimal intersection of 1 bp was required (Figure S6A - left panel). By that criterion, the largest number of overlapping calls with wild mice was found in strains SPRET/EiJ, CAST/EiJ and PWK/PhJ which are derived from *M. m. spretus*, *M. m. castaneus* and *M. m. musculus* subspecies. However, when we normalized the number of overlapping CNVs by dividing it by the average number of detected calls between the two compared individuals, the three strains showed the least similarity to our wild mice samples (Figure S6B - top panel). This is in agreement with their distance to *M. m. domesticus*, and the highest number of overlapping calls with our samples can be explained by the significantly more SVs detected in these strains compared to other strains (Keane et al. 2011). When we applied 50% reciprocal overlap cutoff, wild mouse samples shared on average 998 (median 999) CNVs with inbred strains (Figure S6A - right panel). They shared the largest number of CNVs with WSB/EiJ strain (average 1,337; median 1,471) and also showed highest similarity to it (Figure S6B - lower panel). This is expected, given that the WSB/EiJ is wild derived strain of *M. m. domesticus* subspecies. Overall, most comparisons show unsurprisingly higher similarity to *M. m. domesticus* derived strains, especially to WSB/EiJ and FVB/NJ (Figure S6B).

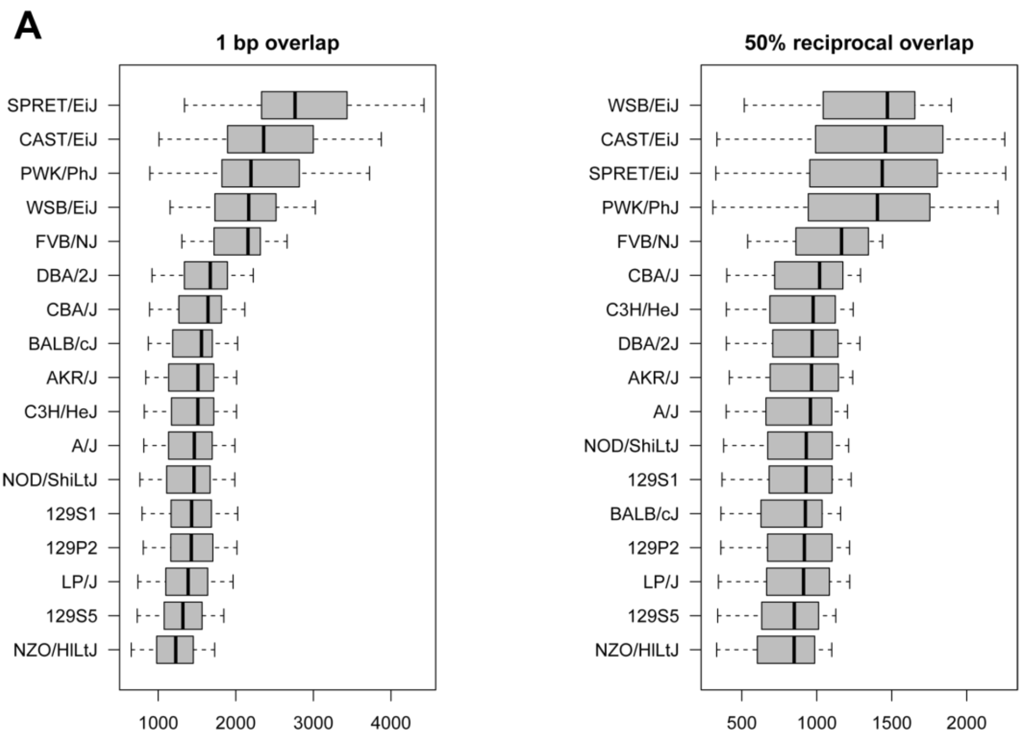

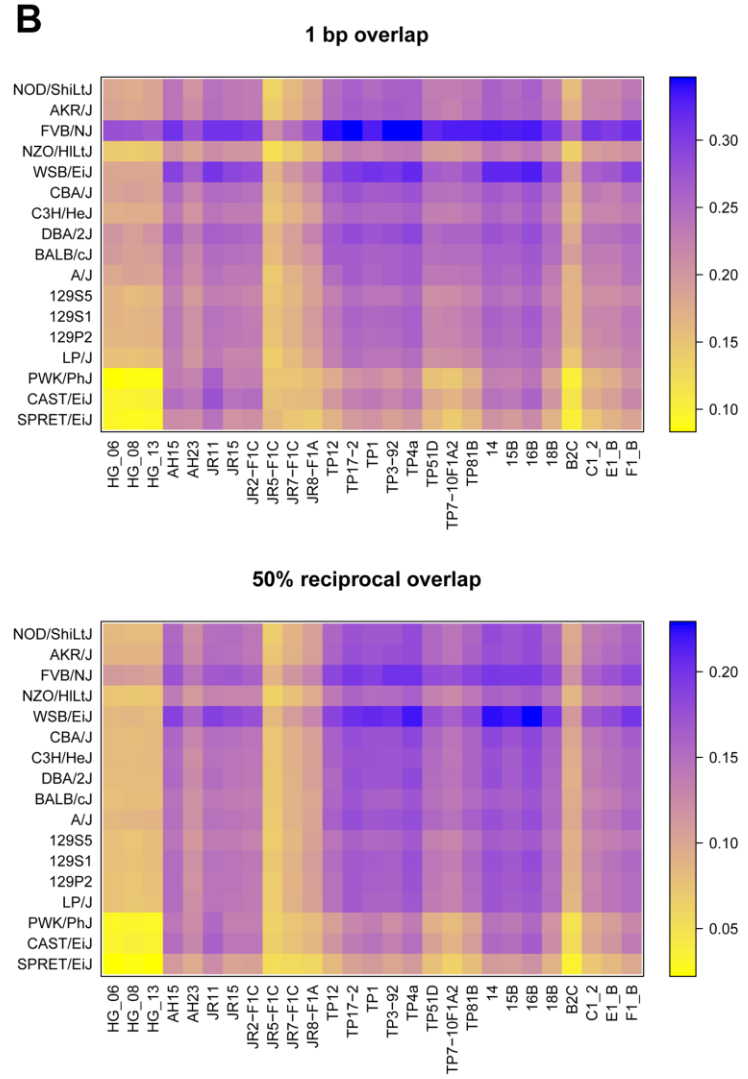

Figure S6. **Comparison with CNVs from inbred mouse strains** studied by Keane et al. 2011 and Wong et al. 2012.
(A) Number of overlapping CNVs between wild mice samples from our study and individual inbred strains of laboratory mice, based on 1 bp minimum intersection (left panel) and minimum 50% reciprocal overlap (right panel).
(B) Similarity matrix based on 1 bp minimum intersection (top panel) and minimum 50% reciprocal overlap (bottom panel), defined as number of overlapping CNVs divided by the average number of detected calls between the two individuals compared. Similarity is presented by a scale, where 0 denotes no similarity and increasing values represent higher similarity.

Text S6: Assessment of CNV detection in regions of high similarity

As a mean of accomplishing uniform depth of coverage across genome, CNVnator keeps reads which can be aligned to multiple locations. This also enables it to detect CNVs in repetitive regions (except for transposable elements - see explanation in Abyzov et al. 2011). In case of paired-end data, CNVnator additionally exploits the information about ends distance and orientation to improve read placement. Moreover, by calculating the fraction of ambiguously mapped reads with zero mapping quality in the called CNV region, CNVnator discards unlikely calls (Abyzov et al. 2011). In order to assess the reliability of calls in repetitive regions, we tested the correlation of genotyped copy numbers of segmental duplications which are annotated at two genomic locations and share over 90% of sequence identity. If each non-uniquely mapping read is simply randomly placed at one of the two possible locations, then it is expected that the two regions to which these reads are mappable will have similar read depth and thus have the same estimated copy number. We genotyped in total 5,517 large SDs (≥10 kb) that are completely encompassed within CNV calls in one individual and compared their copy numbers at alternative locations (Figure S7A). We observe only weak correlation (r = 0.29) between the two locations' copy number, and the large majority of highly discordant values strongly deviated from line of equality (y = x). This shows that CNVnator is able to distinguish similar genomic regions such as SDs.

Given that CNV calling in SDs depends on sequence similarity and can be influenced by sequencing quality, potential artifacts could lead to erroneous conclusions. In our samples, on average 16% (minimum 14%; maximum 19%) of all CNVs overlapped SDs with 98% or more sequence identity. Proportion of such CNVs which also overlapped any part of a gene ranged between 4,5% and 7,2% (5,5% on average). Given these substantial fractions, we tested whether our major findings change when CNVs intersecting highly similar SDs (with ≥ 98% sequence identity) are excluded from analysis. With such data, we repeated the analysis of CNV frequency like the one presented in Figure 2A, and compared the distribution between the original data and data depleted of CNVs intersecting highly similar SDs. We find no significant differences between the two in either overlapping SDs set (Wilcoxon rank sum test; p = 0.576) or non-overlapping SDs set (Wilcoxon rank sum test; p = 0.982). We have also tested how the exclusion of CNV genes intersecting highly similar SDs influences observed patterns of genetic relationship shown in Figure 4. We calculated the Euclidean distance matrix from standardized copy numbers of 1,104 CNV genes which did not intersect highly similar SDs, and compared it to the distance matrix calculated from the original CNV gene dataset by using Mantel test in R package "vegan". Mantel statistics based on Pearson's product-moment correlation revealed strong and significant correlation between the two datasets (r = 0.895; significance = 0.001). These analyses show that although highly similar SDs overlap considerable proportion of CNVs in our dataset, overall conclusions are not changed when they are excluded from analysis, suggesting no major artifacts caused by sequence similarity.

Similarly, potential mis-mapping of the reads could distort CNV calling at pseudogenes and their functional paralogs (parent genes). We picked out seven pseudogenes from our CNV genes list that had one-to-one relationship with the corresponding parent gene, such that, for each case there were no additional paralogs annotated in the RefSeqGene list (see suppl. Table S11 for a list pseudogenes and related parent genes). In all individuals where

these pseudogenes were found completely inside CNV, we genotyped them and their related parent genes. There was no correlation between the copy numbers of pseudogenes with their parent genes, *i.e.* the parent gene had two copies regardless of the pseudogene copy number (Figure S7B). This again shows that, despite high sequence similarity, CNVnator is able to discern different genomic locations.

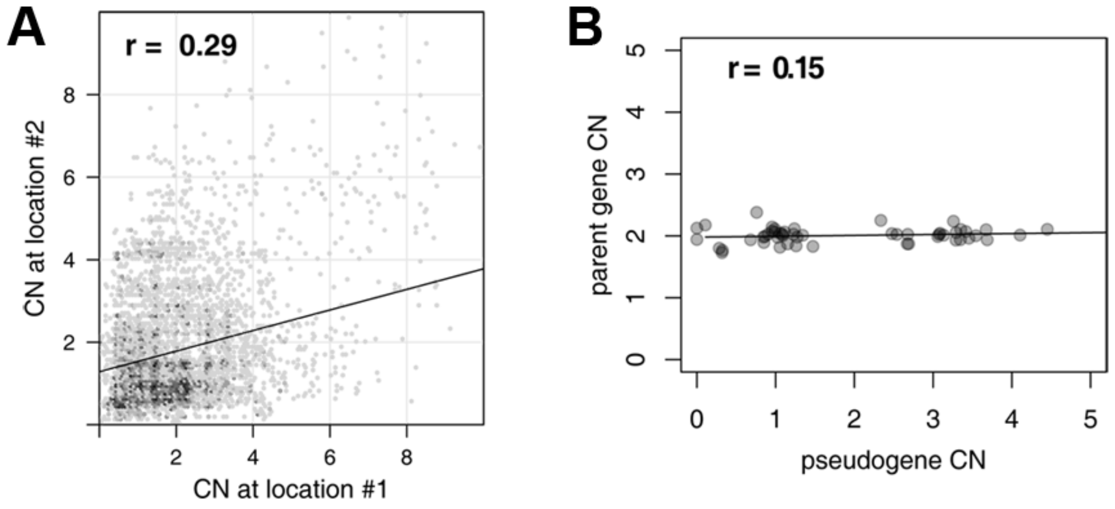

Figure S7. Assessment of CNV detection in regions of high similarity. (A) In total 5,517 large SDs were genotyped in one randomly chosen individual (15B). Each dot represents copy numbers of the two annotated genomic locations per segmental duplication, plotted against each other. Weak correlation and the best fit line strongly deviating from identity line (y=x) illustrate CNVnator's power to discern regions of high similarity. **(B)** No correlation between copy number of pseudogenes and corresponding parent genes was observed. *r* is Pearson's correlation coefficient.

Text S7: CNVR size *vs.* frequency

CNV size and presence in population depends significantly on their overlap with large SDs: CNVs in regions not overlapping SDs are generally smaller and less frequent (Figure 2, main text). This could indicate relatively stronger selective constraints on such CNVs but could also simply reflect size distribution, *i.e.* larger CNVs are more likely to overlap between individuals. To test if the latter is the case, we plotted the CNVR size against their presence in individuals (Figure S8). We found only weak correlation between the two (Pearson's $r = 0.24$; $p < 2.2e^{-16}$), indicating that the observed CNVR frequency is not a technical artifact reflecting the size distribution.

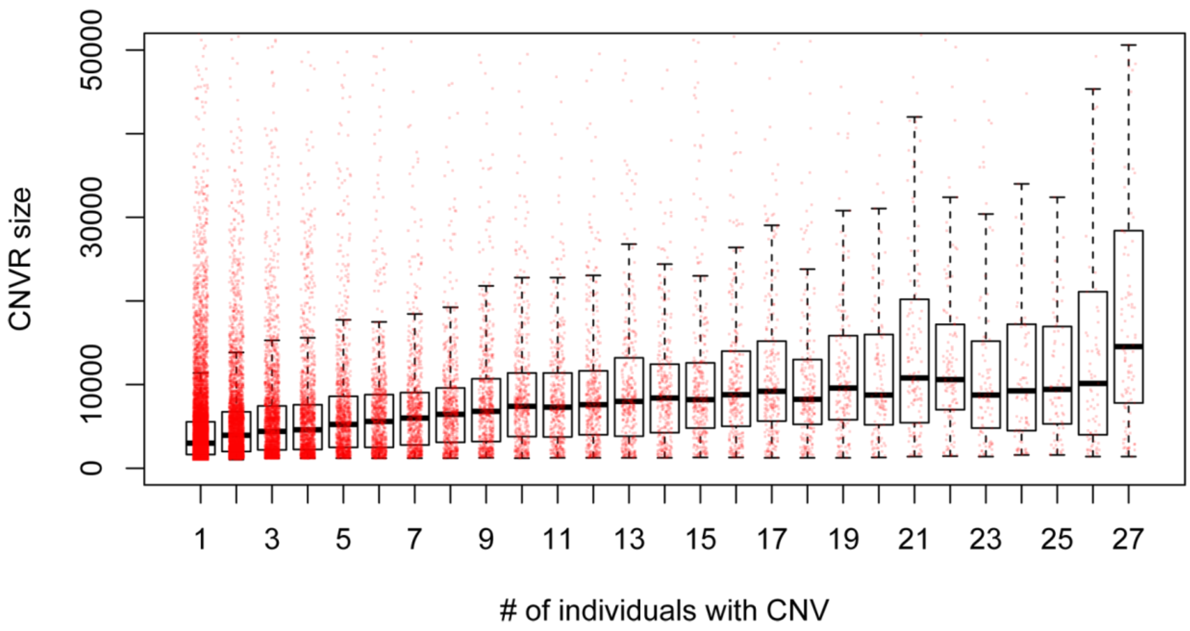

Figure S8. **Distribution of CNVR size per presence in x number of individuals.** Median of distribution is indicated by central line in a box while the edges represent the first and third quartiles. The actual CNVR sizes are overplotted in red: each dot represents one of 28,375 CNVRs that do not overlap large segmental duplications.

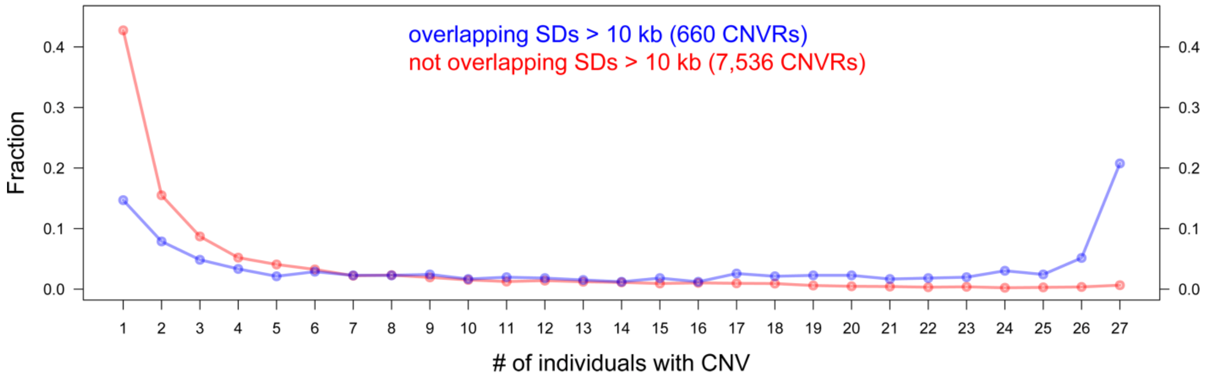

Figure S9. CNVRs intersecting with genes and large segmental duplications are mainly present in multiple samples. Overlapping calls from all individuals were merged into CNVRs and analyzed separately based on their intersection with SDs > 10 kb. Only CNVRs that overlapped at least one gene by any number of nucleotides were considered. Number of individuals with CNV call within each CNVR was counted. There were in total 662 unique CNVRs overlapping SDs (blue) and 7,536 CNVRs not overlapping SDs (red). The graph shows frequencies of CNVR presence across all samples.

Text S8: Size of neural genes and its influence on enrichment at CNVRs

In our set of 28,375 CNVRs that did not overlap large SDs we observe substantial enrichment for terms associated with neurological functions, such as synaptic transmission, nervous system development, learning or memory etc. (Table S3). We asked whether this enrichment was simply a consequence of the large size of genes that are related to these terms.

For GO Term IDs related to neurological functions that were significantly over-represented in our dataset (Table S3; GO:0044708, GO:0007268, GO:0007610, GO:0008344, GO:0030534, GO:0050890, GO:0007270, GO:0035249, GO:0050804, GO:0010975, GO:0007399, GO:0007611, GO:0097090, GO:0050808, GO:0050773, GO:0050806), we extracted all associated genes for *Mus musculus* taxon from http://amigo.geneontology.org. We retained 2,924 non-reduntant gene coordinates which were relevant for mm9 RefSeq gene set. Compared to the rest of the RefSeq genes, these neurologically associated genes are more than twice as large (median 30,9 kbp compared to 14,4 kbp; on average 87,2 kbp compared to 38,4 kbp; Kolmogorov-Smirnov test: $p < 2.2e^{-16}$), similarly to what was previously reported for human genes (Raychaudhuri et al. 2010). We find 26% of them to be overlapped by CNVRs (756/2,924), as opposed to 18% of genes with other functions (4,245/23,832).

To test if arbitrarily positioned genomic fragments preferentially overlap genes with neurological functions, we randomly placed 28,375 non-overlapping segments of matching size as CNVRs in real data, making sure that, as in real data, they are outside of annotated gaps and SDs > 10 kb. We created 10,000 such sets of permuted CNVRs and in each counted the number of neurologically associated genes and other genes overlapped by simulated CNVRs. On average, much larger fraction in both gene groups was affected in simulated data than in observed data: about 45% (1,314/2,924) of neurologically associated genes and 30% (7,135/23,832) of other genes. The fact that fewer genes are overlapped by CNVRs in real data versus in simulated data, suggests that CNVs are biased away from genes, as shown previously (Conrad et al. 2006; Redon et al. 2006). When comparing the affected fractions of neurologically associated genes to other genes, we find that the ratio between the two is significantly smaller in real data (1.44 or 26% to 18%) than in simulated data (1.5 on average or 45% to 30%), indicating that neurologically associated genes are affected by copy number variation less than it is expected by chance. We calculate that the probability of obtaining the ratio of 1.44 or smaller is 0.0087 (87 in 10,000 simulations; Figure S10). This suggests that, despite their substantially larger size, genes associated with neurological functions are less likely to be overlapped by CNVRs than it is expected by chance, even when the bias against copy number variation in genes is taken into account.

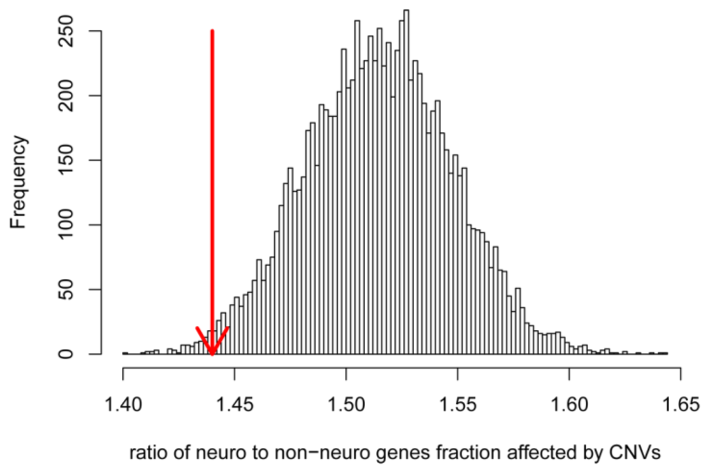

Figure S10. Distribution of calculated ratios for fractions of neurologically functioning genes to other genes that are overlapped by CNVRs in simulated data. Calculated ratios are shown for all 10,000 simulations. Red arrow points to the ratio value in real data.

Text S9: Assessment of genotyping accuracy

To assess the extent to which genotyping accuracy is affected by depth of coverage, we performed a sequential down sampling of reads on one Heligoland individual (HG_08). By using SAMtools (Li et al. 2009) and a custom perl script, we randomly extracted reads from the original sequence file (327,866,406 placed reads in total) in separate subsets of 10%-90% of the reads. In each subset, CNVs were called and CNV genes and their CN determined as described in Methods. The down-sampling experiment was conducted three times independently, each time comparing genotyped CNs between the original file and each subsampled set. We found that calls start to become less reliable only below 100 million reads (Figure S11). Given that our individuals have 3 to 7-times this critical number of reads, we conclude that the gene content and polymorphism analysis is robust and that the genotyping accuracy does not diminish with reduced coverage.

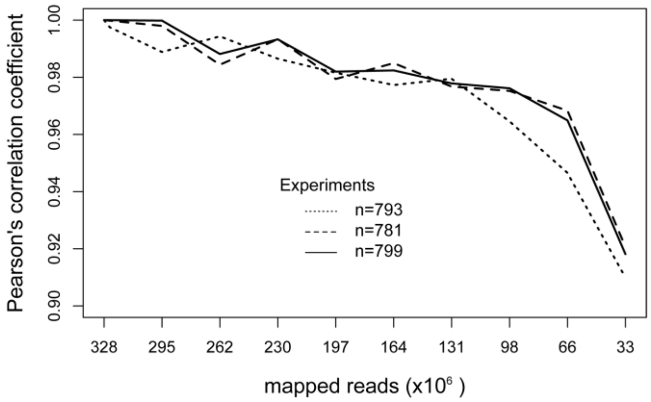

Figure S11. Genotyping accuracy of CNV genes is not drastically affected by depth of coverage. 327,866,406 reads from the original sample were sequentially down-sampled by 10%. Inferred copy numbers were compared between each subsample and the full sample. Computed Pearson's correlation coefficients for each comparison are plotted against each subset of reads including the original set. Three independent down-sampling experiments are shown as different lines. *n* is the number of CNV genes detected and genotyped.

Text S10: Validation of deleted genes analysis

In order to test if the amount of lost genes in HEL population is significantly higher than in mainland populations, we need to account for the smaller sample size. To this end, we created all possible combinations (56) of only three individuals in each mainland population. In each combination we counted the number of autosomal genes which appear to be deleted in all three individuals (normalized RD < 0.5 per diploid). In all three populations, number of deleted genes was considerably higher when only three individuals were considered instead of all eight (median 8.5, 27.5 and 34.5 versus 1, 6 and 16; see Figure S12 and main text); nevertheless, it was still much lower than in HEL population (Figure S12).

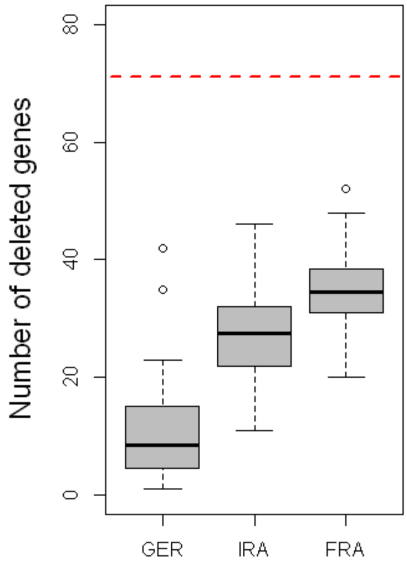

Figure S12. Validation of deleted genes analysis. Larger amount of lost genes detected in HEL population is not an artifact of small sample size. To show this, number of deleted genes was counted in all possible combinations of only three mainland individuals. For each population, distribution of that number across all combinations (n=56) is shown as boxplot. In all combinations of mainland comparisons, number of deleted genes was considerably smaller than in HEL population (71 genes; red dashed line).

Figure S13 - Read depth at CNVs encompassing *Cwc22*, *Hjurp* and *Sfi1*

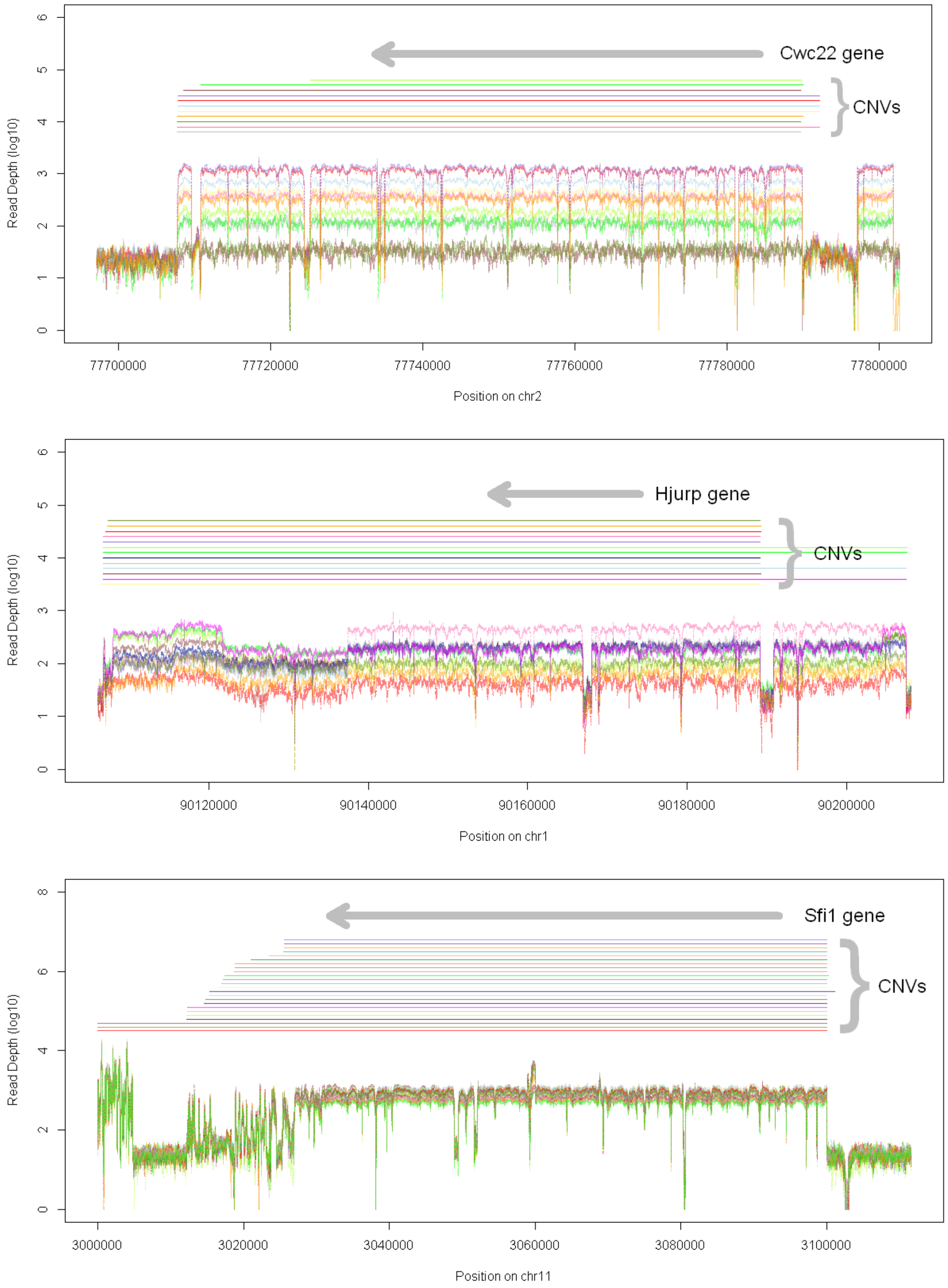

Figure S13. Read depth at CNVs encompassing *Cwc22*, *Hjurp* and *Sfi1*. Read depth signals suggest breakpoints at same locations in multiple individuals. Read depth per base position is shown for each individual CNV and its flanking region in different color. Individual predicted CNVs are shown as bars in corresponding color above the read depth signal. Gray arrows represent gene positions.

Text S11: Validation of V_{ST} analysis

To test how the difference in sample size between Heligoland mice (n=3) and the other three populations (n=8 per population) influences the results of V_{ST} statistics (Table S7), we repeated the analysis on subsets of mainland samples. For all possible combinations (56) made of just three individuals from one population with all eight individuals from the other, mean V_{ST} values were calculated and compared across all population pairs and combination groups. In all resulting comparisons, the mean V_{ST} was on average significantly lower than in any comparison with the HEL sample (Figure S14). In addition, the values were similar to the original eight-to-eight comparisons, providing strong support for the observed higher differentiation of the HEL population.

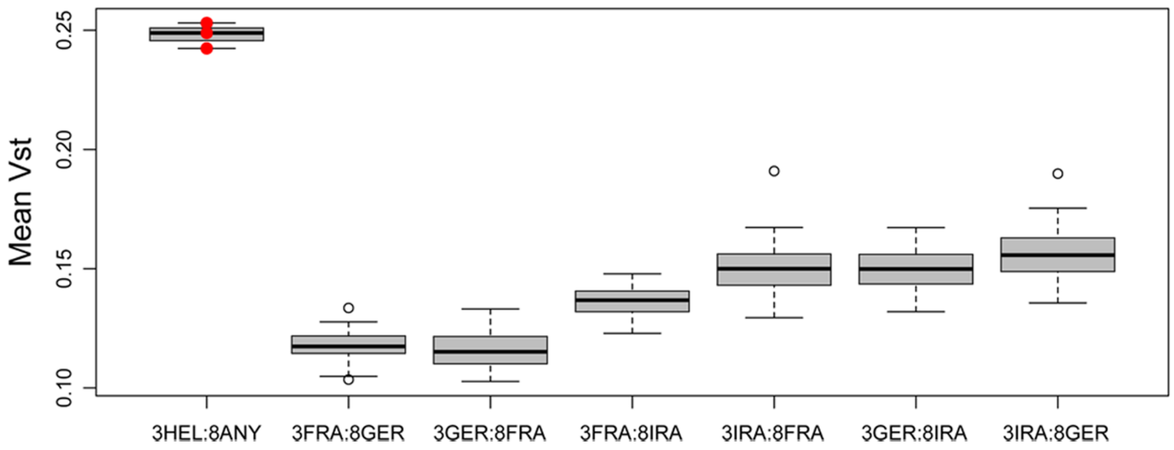

Figure S14. Validation of V_{ST} analysis. Much higher differentiation of Heligoland mice from mainland populations is not an artifact of small sample size. To show this, V_{ST} values were computed for all possible combinations of 3:8 mainland individuals and mean V_{ST} was calculated for each. In all combinations of mainland comparisons, V_{ST} was on average significantly lower than in any comparison with Heligoland sample.

Text S12: Outlier analysis

In order to detect genes with particularly large average copy number differences between populations, we performed pairwise comparisons of average copy number per population for all 1,863 CNV genes (Figure S15A). Outliers were identified as points that are at least 4 standard deviations (equivalent to p = 0.001) away from the best fit line of the resulting distribution. For the comparisons with HEL mice, the distribution was too spread out to detect any outliers (not shown). In the other three comparisons we detected in total 15 outliers (red dots in Figure S15A) corresponding to 13 genes (Figure S15B). Many of these are polymorphic within populations, *i.e.* individuals of the population can contain very different numbers of such genes.

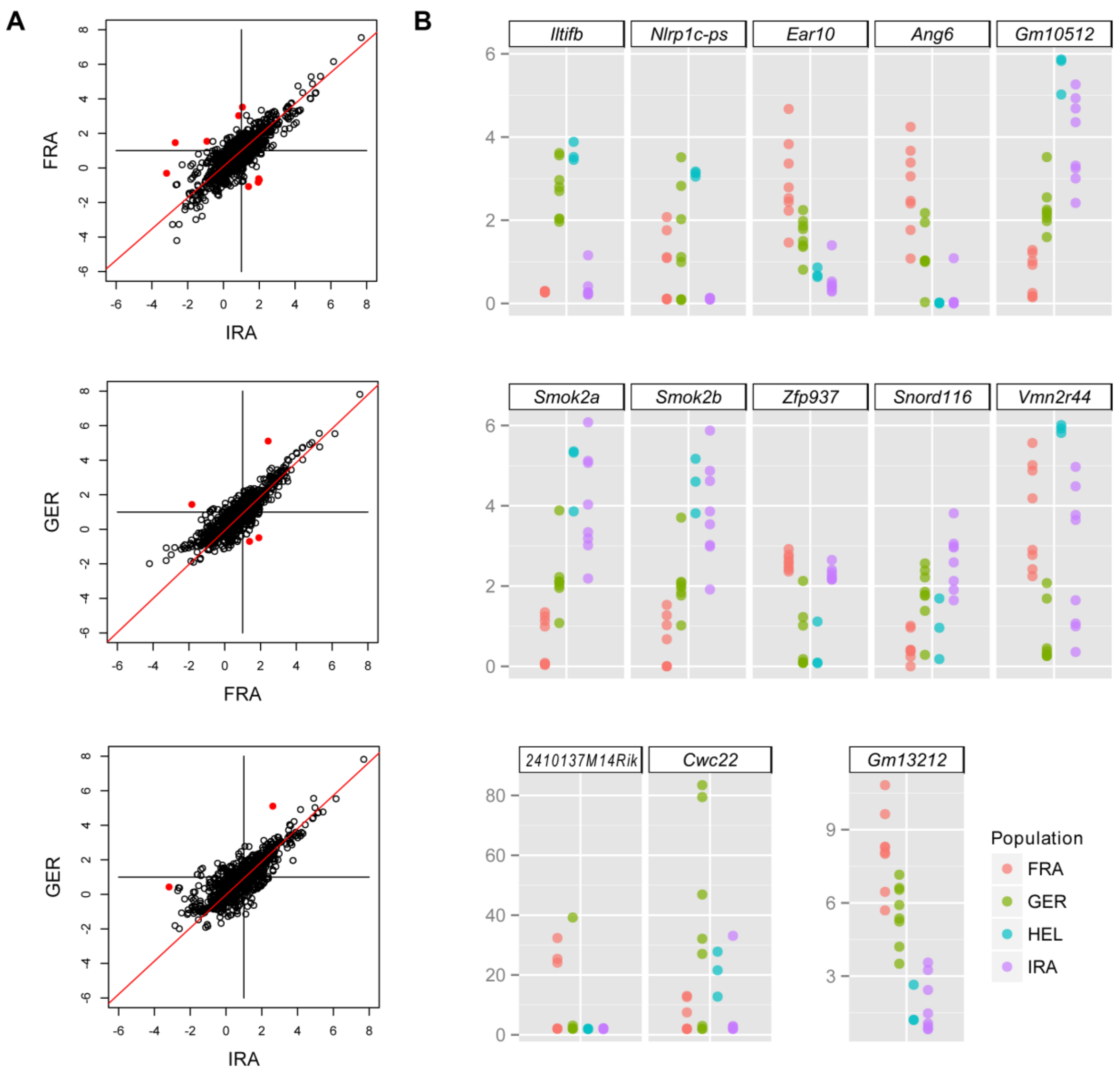

Figure S15. Differences in population average gene copy number. (A) $\log_2$ transformed values of mean copy number (per diploid genome) for each CNV gene were plotted between populations. The most differentiated genes are identified as points (red dots) which are a minimum of 4 standard deviations away from the best fit line of the resulting distribution (red line). Their individual copy numbers are plotted in panel **(B)**.

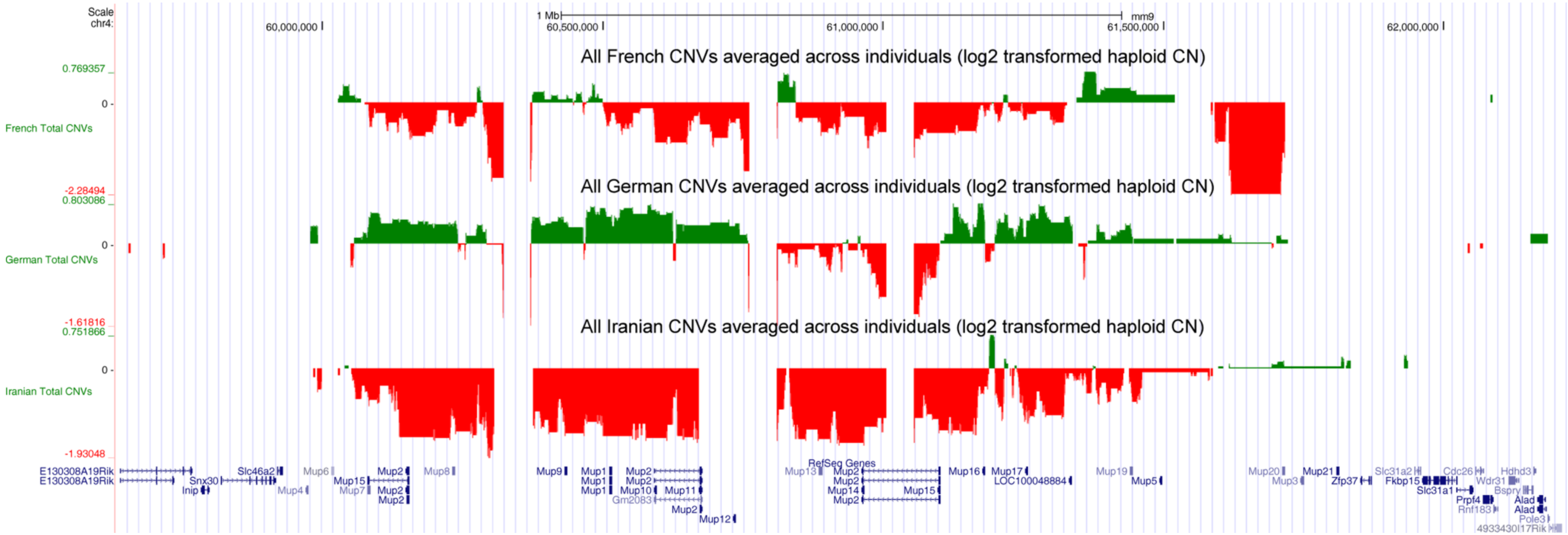

Figure S16. UCSC Genome Browser view of CNVs in the major urinary protein locus of the three mainland populations. Each track represents one population. CNVs are shown as histograms, where the bar height is proportional to average copy number of the eight individuals. Positive bars are green (duplications), negative are red (deletions). Values are per haploid, log2 transformed.

Table S1. Read mapping and CNV discovery statistics

Population	Individual	Mapped (after PCR duplicate removal)	Average coverage (fold) §	All CNVs	Singleton CNVs	Average CNV length	Genic CNVs	CNV genes
**IRA**	AH15	647,577,438	23.29	8,302	563	10,474	281	603
	AH23	728,625,638	26.21	9,878	1,194	9,321	312	625
	JR11	702,963,516	25.28	10,714	952	9,103	314	663
	JR15	654,362,227	23.54	8,198	498	10,756	331	659
	JR2-F1C	675,549,496	24.30	8,853	823	10,144	293	617
	JR5-F1C	532,452,937	19.15	7,099	1,426	15,294	390	725
	JR7-F1C	571,560,621	20.56	6,100	489	13,572	310	667
	JR8-F1A	527,406,654	18.97	5,477	402	14,261	305	659
	**median**	**650,969,832.5**	**23.41**	**8,250.0**	**693.0**	**10,615**	**311.0**	**659.0**
	**average**	**630,062,315.9**	**22.66**	**8,077.6**	**793.4**	**11,616**	**317.0**	**652.3**
	**overlapping**			**2,576 ± 657***				
**FRA**	14	693,974,968	24.96	7,540	246	11,014	269	598
	15B	671,761,851	24.16	7,277	194	10,661	254	543
	16B	703,427,526	25.30	7,674	277	10,711	265	567
	18B	576,102,049	20.72	6,032	143	12,116	231	544
	B2C	438,203,162	15.76	3,905	91	16,278	247	594
	C1_2	569,548,403	20.49	5,138	134	14,605	256	637
	E1_B	630,754,637	22.69	6,541	174	11,513	250	542
	F1_B	656,403,130	23.61	7,518	269	10,426	264	572
	**median**	**643,578,883.5**	**23.15**	**6,909.0**	**184.0**	**11,264**	**255.0**	**569.5**
	**average**	**617,521,965.8**	**22.21**	**6,453.1**	**191.0**	**12,165**	**254.5**	**574.6**
	**overlapping**			**2,759 ± 624***				
**GER**	TP12	630,227,518	22.67	6,218	208	12,416	259	573
	TP17-2	675,001,442	24.28	7,570	315	10,020	245	535
	TP1	645,368,343	23.21	7,618	338	10,181	243	520
	TP3-92	669,449,227	24.08	7,257	256	10,354	248	509
	TP4a	691,555,207	24.87	7,822	337	10,197	253	552
	TP51D	591,442,966	21.27	6,121	213	11,889	245	572
	TP7-10F1A2	572,339,487	20.59	5,200	140	13,262	243	579
	TP81B	630,369,818	22.67	6,030	204	11,720	214	484
	**median**	**637,869,080.5**	**22.94**	**6,737.5**	**234.5**	**11,037**	**245.0**	**543.5**
	**average**	**638,219,251.0**	**22.96**	**6,729.5**	**251.4**	**11,255**	**243.8**	**540.5**
	**overlapping**			**2,664 ± 291***				
**HEL**	HG_06	283,535,524	10.20	3,714	150	17,658	238	538
	HG_08	318,549,500	11.46	3,707	175	16,501	209	453
	HG_13	313,745,605	11.28	3,722	201	17,372	224	543
	**median**	**313,745,605.0**	**11.28**	**3,714.0**	**175.0**	**17,372**	**224.0**	**538.0**
	**average**	**305,276,876.3**	**10.98**	**3,714.3**	**175.3**	**17,177**	**223.7**	**511.3**
	**overlapping**			**2,025 ± 56***				

§ Coverage was calculated as number of mapped reads multiplied by average read length and divided by genome size
* Mean ± standard deviation

Table S2. GO term enrichment analysis of genes in CNVRs overlapping SDs > 10 kb

GO Term	Description	P-value	FDR q-value	Enrichment	total # of genes	total # of genes associated with a specific GO term	# of genes in the target set	# of genes in the intersection
GO:0019882	antigen processing and presentation	4.85E-12	5.76E-08	5.15	21317	83	1247	25
GO:0002474	antigen processing and presentation of peptide antigen via MHC class I	8.57E-12	5.08E-08	7.16	21317	43	1247	18
GO:0007186	G-protein coupled receptor signaling pathway	5.04E-11	1.99E-07	1.6	21317	1992	1247	186
GO:0048002	antigen processing and presentation of peptide antigen	4.88E-10	1.45E-06	5.51	21317	59	1247	19
GO:2001015	negative regulation of skeletal muscle cell differentiation	1.46E-09	3.47E-06	12.82	21317	12	1247	9
GO:0006959	humoral immune response	3.25E-09	6.43E-06	4.18	21317	94	1247	23
GO:0035458	cellular response to interferon-beta	2.57E-07	4.35E-04	7.43	21317	23	1247	10
GO:0050909	sensory perception of taste	3.24E-07	4.81E-04	4.21	21317	69	1247	17
GO:2001014	regulation of skeletal muscle cell differentiation	7.27E-07	9.59E-04	7.69	21317	20	1247	9
GO:0042742	defense response to bacterium	1.03E-06	1.22E-03	2.75	21317	174	1247	28
GO:0009617	response to bacterium	1.14E-06	1.23E-03	2.63	21317	195	1247	30
GO:0035456	response to interferon-beta	2.25E-06	2.23E-03	6.11	21317	28	1247	10
GO:0043330	response to exogenous dsRNA	2.92E-06	2.67E-03	4.27	21317	56	1247	14
GO:0033141	positive regulation of peptidyl-serine phosphorylation of STAT protein	3.26E-06	2.76E-03	5.89	21317	29	1247	10
GO:0033139	regulation of peptidyl-serine phosphorylation of STAT protein	3.26E-06	2.58E-03	5.89	21317	29	1247	10
GO:0002323	natural killer cell activation involved in immune response	6.48E-06	4.80E-03	5.51	21317	31	1247	10
GO:0045343	regulation of MHC class I biosynthetic process	8.93E-06	6.23E-03	5.34	21317	32	1247	10
GO:0043331	response to dsRNA	1.28E-05	8.45E-03	3.8	21317	63	1247	14
GO:0002250	adaptive immune response	4.56E-05	2.85E-02	3.11	21317	88	1247	16

Table S3. GO term enrichment analysis of genes in CNVRs not overlapping SDs > 10 kb

GO Term	Description	P-value	FDR q-value	Enrichment	total # of genes	total # of genes associated with a specific GO term	# of genes in the target set	# of genes in the intersection
GO:0022610	biological adhesion	2.40E-13	2.85E-09	1.59	21317	680	4186	212
GO:0007155	cell adhesion	2.84E-13	1.69E-09	1.59	21317	673	4186	210
GO:0044763	single-organism cellular process	5.40E-08	2.14E-04	1.1	21317	7554	4186	1632
GO:0051056	regulation of small GTPase mediated signal transduction	5.58E-08	1.66E-04	1.85	21317	190	4186	69
GO:0070838	divalent metal ion transport	2.21E-07	5.24E-04	1.76	21317	211	4186	73
GO:0007626	locomotory behavior	2.30E-07	4.55E-04	1.82	21317	185	4186	66
GO:0033124	regulation of GTP catabolic process	2.32E-07	3.93E-04	1.59	21317	340	4186	106
GO:0043087	regulation of GTPase activity	3.20E-07	4.75E-04	1.58	21317	338	4186	105
GO:0030001	metal ion transport	6.24E-07	8.23E-04	1.45	21317	516	4186	147
GO:0007215	glutamate receptor signaling pathway	6.55E-07	7.78E-04	2.89	21317	37	4186	21
GO:0044708	single-organism behavior	6.88E-07	7.42E-04	1.55	21317	351	4186	107
GO:0006816	calcium ion transport	1.07E-06	1.05E-03	1.75	21317	192	4186	66
GO:0070588	calcium ion transmembrane transport	1.82E-06	1.66E-03	1.96	21317	117	4186	45
GO:0072511	divalent inorganic cation transport	2.86E-06	2.43E-03	1.66	21317	224	4186	73
GO:0034765	regulation of ion transmembrane transport	4.30E-06	3.40E-03	1.57	21317	282	4186	87
GO:0007268	synaptic transmission	4.52E-06	3.35E-03	1.73	21317	180	4186	61
GO:0016337	cell-cell adhesion	5.07E-06	3.54E-03	1.57	21317	275	4186	85
GO:0007610	behavior	5.09E-06	3.36E-03	1.42	21317	487	4186	136
GO:0046578	regulation of Ras protein signal transduction	5.29E-06	3.30E-03	1.77	21317	158	4186	55
GO:0009118	regulation of nucleoside metabolic process	5.59E-06	3.31E-03	1.48	21317	374	4186	109
GO:0030811	regulation of nucleotide catabolic process	6.52E-06	3.68E-03	1.48	21317	371	4186	108
GO:0033121	regulation of purine nucleotide catabolic process	6.52E-06	3.51E-03	1.48	21317	371	4186	108
GO:0007156	homophilic cell adhesion	6.78E-06	3.50E-03	2.16	21317	73	4186	31
GO:0008344	adult locomotory behavior	7.81E-06	3.86E-03	2	21317	94	4186	37
GO:0006493	protein O-linked glycosylation	8.90E-06	4.22E-03	2.78	21317	33	4186	18
GO:0006812	cation transport	9.66E-06	4.41E-03	1.34	21317	684	4186	180
GO:0009187	cyclic nucleotide metabolic process	1.34E-05	5.90E-03	2.35	21317	52	4186	24
GO:0030534	adult behavior	1.57E-05	6.66E-03	1.75	21317	148	4186	51
GO:0034762	regulation of transmembrane transport	1.91E-05	7.83E-03	1.52	21317	292	4186	87
GO:0030032	lamellipodium assembly	2.33E-05	9.21E-03	2.81	21317	29	4186	16
GO:0046058	cAMP metabolic process	2.33E-05	8.92E-03	2.81	21317	29	4186	16
GO:1900542	regulation of purine nucleotide metabolic process	2.44E-05	9.05E-03	1.39	21317	484	4186	132
GO:0006468	protein phosphorylation	3.04E-05	1.09E-02	1.33	21317	648	4186	169
GO:0006464	cellular protein modification process	3.20E-05	1.12E-02	1.19	21317	1750	4186	409
GO:0036211	protein modification process	3.20E-05	1.08E-02	1.19	21317	1750	4186	409
GO:0031344	regulation of cell projection organization	3.23E-05	1.06E-02	1.45	21317	350	4186	100
GO:0006811	ion transport	3.27E-05	1.05E-02	1.26	21317	1004	4186	248
GO:0016310	phosphorylation	3.88E-05	1.21E-02	1.27	21317	920	4186	229
GO:0006140	regulation of nucleotide metabolic process	4.12E-05	1.25E-02	1.37	21317	489	4186	132
GO:0032318	regulation of Ras GTPase activity	4.18E-05	1.24E-02	1.56	21317	232	4186	71
GO:0043412	macromolecule modification	5.21E-05	1.51E-02	1.18	21317	1844	4186	427
GO:0006793	phosphorus metabolic process	5.26E-05	1.49E-02	1.18	21317	1882	4186	435
GO:2001015	negative regulation of skeletal muscle cell differentiation	5.31E-05	1.46E-02	3.82	21317	12	4186	9
GO:0006796	phosphate-containing compound metabolic process	5.33E-05	1.44E-02	1.18	21317	1835	4186	425
GO:0050890	cognition	5.94E-05	1.57E-02	1.59	21317	198	4186	62
GO:0030030	cell projection organization	6.00E-05	1.55E-02	1.34	21317	576	4186	151
GO:0018210	peptidyl-threonine modification	6.06E-05	1.53E-02	2.37	21317	43	4186	20
GO:0007270	neuron-neuron synaptic transmission	7.52E-05	1.86E-02	2.24	21317	50	4186	22
GO:0051049	regulation of transport	8.68E-05	2.10E-02	1.22	21317	1235	4186	295
GO:0035249	synaptic transmission, glutamatergic	1.08E-04	2.57E-02	2.74	21317	26	4186	14
GO:0050804	regulation of synaptic transmission	1.23E-04	2.86E-02	1.53	21317	227	4186	68
GO:0051179	localization	1.24E-04	2.82E-02	1.39	21317	400	4186	109
GO:0097503	sialylation	1.33E-04	2.97E-02	3.11	21317	18	4186	11
GO:0010975	regulation of neuron projection development	1.39E-04	3.06E-02	1.46	21317	286	4186	82
GO:0019932	second-messenger-mediated signaling	1.43E-04	3.09E-02	1.82	21317	98	4186	35
GO:0006486	protein glycosylation	1.70E-04	3.60E-02	1.68	21317	133	4186	44
GO:0043413	macromolecule glycosylation	1.70E-04	3.54E-02	1.68	21317	133	4186	44
GO:0035556	intracellular signal transduction	1.76E-04	3.59E-02	1.22	21317	1052	4186	253
GO:0032879	regulation of localization	1.77E-04	3.57E-02	1.17	21317	1665	4186	384
GO:0017157	regulation of exocytosis	1.80E-04	3.57E-02	1.8	21317	99	4186	35
GO:0007399	nervous system development	1.87E-04	3.64E-02	1.43	21317	318	4186	89
GO:0007611	learning or memory	2.18E-04	4.16E-02	1.56	21317	186	4186	57
GO:0044765	single-organism transport	2.31E-04	4.36E-02	1.15	21317	2070	4186	468
GO:0043269	regulation of ion transport	2.34E-04	4.34E-02	1.33	21317	485	4186	127
GO:0042391	regulation of membrane potential	2.63E-04	4.80E-02	1.5	21317	220	4186	65
GO:0097090	presynaptic membrane organization	2.77E-04	4.98E-02	3.96	21317	9	4186	7

Table S5. GO term enrichment analysis of CNV genes

GO Term	Description	P-value	FDR q-value	Enrichment	total # of genes	total # of genes associated with a specific GO term	# of genes in the target set	# of genes in the intersection
GO:0007186	G-protein coupled receptor signaling pathway	8.33E-54	1.03E-49	2.47	21365	1990	1331	306
GO:0007606	sensory perception of chemical stimulus	8.86E-50	5.49E-46	2.90	21365	1229	1331	222
GO:0050911	detection of chemical stimulus involved in sensory perception of smell	1.20E-45	4.94E-42	2.94	21365	1091	1331	200
GO:0007608	sensory perception of smell	1.20E-45	3.71E-42	2.90	21365	1128	1331	204
GO:0050907	detection of chemical stimulus involved in sensory perception	3.92E-43	9.72E-40	2.84	21365	1132	1331	200
GO:0009593	detection of chemical stimulus	1.14E-41	2.36E-38	2.77	21365	1157	1331	200
GO:0050906	detection of stimulus involved in sensory perception	8.57E-41	1.52E-37	2.73	21365	1182	1331	201
GO:0051606	detection of stimulus	3.00E-37	4.65E-34	2.57	21365	1268	1331	203
GO:0007600	sensory perception	6.07E-36	8.36E-33	2.37	21365	1521	1331	225
GO:0050877	neurological system process	8.24E-27	1.02E-23	2.04	21365	1798	1331	229
GO:0007166	cell surface receptor signaling pathway	1.74E-23	1.96E-20	1.68	21365	3152	1331	330
GO:0003008	system process	2.69E-20	2.78E-17	1.81	21365	2084	1331	235
GO:0002474	antigen processing and presentation of peptide antigen via MHC class I	1.07E-15	1.02E-12	7.69	21365	48	1331	23
GO:0048002	antigen processing and presentation of peptide antigen	2.16E-15	1.91E-12	6.52	21365	64	1331	26
GO:0019882	antigen processing and presentation	1.06E-10	8.79E-08	4.35	21365	96	1331	26
GO:2001015	negative regulation of skeletal muscle cell differentiation	2.55E-09	1.98E-06	12.04	21365	12	1331	9
GO:0042221	response to chemical	5.94E-09	4.33E-06	1.37	21365	3373	1331	287
GO:0033141	positive regulation of peptidyl-serine phosphorylation of STAT protein	6.26E-08	4.31E-05	6.64	21365	29	1331	12
GO:0033139	regulation of peptidyl-serine phosphorylation of STAT protein	6.26E-08	4.08E-05	6.64	21365	29	1331	12
GO:0002323	natural killer cell activation involved in immune response	1.51E-07	9.38E-05	6.21	21365	31	1331	12
GO:0045343	regulation of MHC class I biosynthetic process	2.28E-07	1.35E-04	6.02	21365	32	1331	12
GO:0019236	response to pheromone	1.45E-06	8.16E-04	3.24	21365	104	1331	21
GO:0006959	humoral immune response	2.00E-06	1.08E-03	3.18	21365	106	1331	21
GO:2001014	regulation of skeletal muscle cell differentiation	2.04E-06	1.05E-03	6.88	21365	21	1331	9
GO:0007165	signal transduction	7.68E-06	3.81E-03	1.23	21365	4388	1331	337
GO:0050909	sensory perception of taste	1.02E-05	4.84E-03	3.47	21365	74	1331	16
GO:0002250	adaptive immune response	1.08E-05	4.98E-03	3.18	21365	91	1331	18
GO:0042100	B cell proliferation	2.78E-05	1.23E-02	4.01	21365	48	1331	12
GO:0042742	defense response to bacterium	3.75E-05	1.60E-02	2.28	21365	197	1331	28
GO:0030101	natural killer cell activation	6.54E-05	2.70E-02	3.70	21365	52	1331	12
GO:0009617	response to bacterium	9.31E-05	3.72E-02	2.14	21365	218	1331	29
GO:0043330	response to exogenous dsRNA	1.17E-04	4.53E-02	3.50	21365	55	1331	12

Table S9. Diploid copy number of genes in amylase cluster by individual*

		FRA								IRA								GER								HEL		
**Gene Symbol**	**Location (mm9)**	**14**	**15B**	**16B**	**18B**	**B2C**	**C1_2**	**E1_B**	**F1_B**	**AH15**	**AH23**	**JR11**	**JR15**	**JR2-F1C**	**JR5-F1C**	**JR7-F1C**	**JR8-F1A**	**TP81B**	**TP7-10F1A2**	**TP51D**	**TP4a**	**TP3-92**	**TP17-2**	**TP12**	**TP1**	**HG_06**	**HG_08**	**HG_13**
Amy2a2	chr3:113082754-113094367	3	2	3	3	3	3	2	2	1	2	1	1	1	1	1	1	2	2	2	2	2	2	2	2	1	1	1
	chr3:113115366-113126970	3	2	3	3	3	3	2	2	1	2	1	1	1	1	1	1	2	2	2	2	2	2	2	2	1	1	1
	chr3:113147966-113159576	3	2	3	3	2	2	2	2	1	2	1	1	1	1	1	1	2	2	2	2	2	2	2	2	1	1	1
Amy2a5	chr3:113052095-113061617	2	2	3	2	2	2	2	2	1	1	1	1	1	1	1	1	2	2	2	2	2	2	2	2	1	1	1
	chr3:113084762-113094229	3	2	3	2	2	2	2	2	1	2	1	1	1	1	1	1	2	2	2	2	2	2	2	2	1	1	1
	chr3:113117374-113126832	3	2	3	3	3	3	2	2	1	2	1	1	1	1	1	1	2	2	2	2	2	2	2	2	1	1	1
	chr3:113149974-113159438	2	2	3	2	2	2	2	2	1	2	1	1	1	1	1	1	2	2	2	2	2	2	2	2	1	1	1

***** Rounded off copy numbers from the CNVnator's genotyping output are shown

Table S10. Diploid copy number of genes in *Mup* cluster by individual*

Gene Symbol	Location (mm9)	FRA								IRA								GER								HEL		
		14	15B	16B	18B	B2C	C1_2	E1_B	F1_B	AH15	AH23	JR11	JR15	JR2-F1C	JR5-F1C	JR7-F1C	JR8-F1A	TP81B	TP7-10F1A2	TP51D	TP4a	TP3-92	TP17-2	TP12	TP1	HG_06	HG_08	HG_13
Mup7	chr4:60079340-60083347	2	2	2	2	2	2	1	2	1	2	1	1	1	1	1	1	3	2	3	1	2	2	2	2	2	2	2
Mup15	chr4:60079340-60152730	1	2	1	1	1	2	1	2	1	2	1	2	1	1	1	1	3	4	5	1	3	2	5	2	3	3	3
Mup2	chr4:60148804-60152729	1	2	1	1	2	2	1	2	1	1	1	1	1	1	1	1	2	4	5	1	3	2	5	2	2	2	2
Mup2	chr4:60149719-60152729	1	1	1	1	2	2	1	2	1	1	1	1	1	1	1	1	2	4	5	1	2	2	4	2	2	2	2
Mup8	chr4:60231492-60235471	2	2	1	2	2	2	2	2	1	1	1	1	1	2	1	1	2	4	5	1	1	2	5	2	2	2	2
Mup9	chr4:60432031-60434824	2	2	2	2	2	2	2	3	1	2	1	2	1	1	1	1	3	4	5	1	3	2	5	2	3	2	3
Mup1	chr4:60510884-60514832	2	2	1	2	2	2	1	2	1	1	1	1	1	1	1	1	2	3	4	1	1	2	4	1	2	2	2
Mup1	chr4:60511805-60514832	2	2	2	2	2	2	1	2	1	1	1	1	1	1	1	1	2	3	5	1	2	2	4	1	3	2	3
Mup10	chr4:60591132-60595027	1	1	1	1	1	2	1	1	1	1	1	1	1	1	1	1	3	5	6	1	2	2	5	2	3	3	3
Mup11	chr4:60671338-60675267	2	1	1	2	2	2	2	3	1	1	1	1	1	2	1	1	4	4	6	0	1	1	4	2	2	2	2
Mup2	chr4:60672252-60675243	2	1	1	2	2	2	1	3	1	1	1	1	1	2	1	1	4	4	6	0	1	1	4	2	2	2	2
Mup12	chr4:60732253-60736153	2	1	1	1	1	2	1	1	1	1	1	1	1	1	1	1	2	3	4	0	1	2	3	1	2	3	2
Mup13	chr4:60885338-60889318	1	1	1	1	1	2	1	2	1	1	1	1	1	0	1	1	1	2	2	2	2	2	2	1	1	1	1
Mup14	chr4:60961068-60965030	2	1	1	2	2	2	1	2	1	1	1	1	1	0	0	1	1	2	3	2	2	2	3	2	1	1	1
Mup15	chr4:61096818-61100736	2	1	1	2	2	2	1	2	1	2	1	1	1	1	1	1	1	1	2	2	2	2	2	2	1	1	1
Mup16	chr4:61176624-61180518	2	1	1	1	1	3	1	2	2	2	2	2	1	1	2	1	3	5	5	1	2	3	5	3	2	2	2
Mup17	chr4:61252962-61256864	2	1	1	2	1	2	1	2	1	1	1	1	1	1	1	1	1	3	2	3	3	3	3	2	1	1	1
Mup19	chr4:61439361-61443258	3	2	2	2	3	3	2	4	2	1	2	2	1	1	1	2	3	4	4	2	3	2	5	2	3	3	3
Mup5	chr4:61492352-61496214	2	2	2	2	2	2	2	4	2	2	2	2	2	1	2	2	2	2	2	2	3	2	2	2	2	2	2
Mup20	chr4:61711268-61715151	1	1	1	1	2	1	1	1	2	3	2	2	2	2	2	2	2	3	2	2	2	2	2	2	2	2	2
Mup3	chr4:61744510-61748346	2	2	2	2	2	2	2	2	2	2	2	2	2	2	2	2	2	2	2	2	2	2	2	2	2	2	2
Mup21	chr4:61808865-61811875	2	2	2	2	2	2	2	2	2	2	2	2	2	2	2	2	2	2	2	2	2	2	2	2	2	2	2

* Rounded off copy numbers from the CNVnator's genotyping output are shown

Table S11: Pseudogene-parent gene pairs used for copy number correlation analysis

pseudogene	mm9 coordinates	parent gene	mm9 coordinates
Alms1-ps2	chr6:85742110-85754051	*Alms1*	chr6:85537524-85652745
Bambi-ps1	chr2:122292318-122293533	*Bambi*	chr18:3507954-3516402
Rpl31-ps12	chr16:16819805-16820289	*Rpl31*	chr1:39424695-39428755
Rps19-ps3	chr4:147195885-147196311	*Rps19*	chr7:25669732-25674821
Smarca5-ps	chr4:145054112-145057864	*Smarca5*	chr8:83223842-83263358
Sycp1-ps1	chr7:19371650-19374763	*Sycp1*	chr3:102622421-102740023
Tubb2a-ps2	chr12:11889001-11889705	*Tubb2a*	chr13:34166146-34169877

Table S12. Assays used for ddPCR

Assay Name	Amplicon Location (NCBI37/mm9)	Primer 1 (5' -> 3')	Primer 2 (5' -> 3')	Probe (5' -> 3')
BC018473	chr11:116615049-116615172	AAT TTG CCA AAA GTT AGT CAG GTT	TCC TTA TGC TTT GGG ATC TAT CAG	/56-FAM/CTG GAA TGC /ZEN/CTC ACG GAA GCC /3IABkFQ/
Bglap3	chr3:88172746-88172857	AAG CAG GGT CAA GCT CAC ATA G	ATA TTA ATG CCA CTG TGT GTT GGT	/56-FAM/TGG GCT CCA /ZEN/GGG GAT CTG /3IABkFQ/
Chil4	chr3:106008103-106008205	GCA CTT TAG GCA TGA GTT CCA	CCA CAT TCC TAC GAG TGC TTG	/56-FAM/TGC TTA ATG /ZEN/GCT GCA AAA TGA ATC AG/3IABkFQ/
Cma1	chr14:56562508-56562594	ATT AAG GAT AAG CAG CGC CTT G	TGC AGT GGC TTC CTG ATA AGA	/56-FAM/CAG TGA GCT /ZEN/GCA GTC AGC ACA A/3IABkFQ/
Cwc22	chr2:77743835-77743912	TAA AGA CTG CTC GCA AAC ACC	CTA ATG CTC TTG GTG GCA CTT	/56-FAM/TGT GGT GTG /ZEN/CAC AAG GGA CG/3IABkFQ/
Defb8	chr8:19445828-19445968	GGT TTG CAG GAT CTT TGT CTT CT	CAA TGA TCC AGT AAC TTA CAT TCG A	/56-FAM/CCT AAG GCC /ZEN/AAT GCA CCG ATA CT/3IABkFQ/
Dux	chr10:57673792-57673928	GAG CAT CCT TAA TAT TGC CGT CAT	ATC AGT CGA CTT AAT GGG AGC TAT	/56-FAM/CCA TGT AAC /ZEN/CAA CTG CAC AGC TCA /3IABkFQ/
Glo1	chr17:30745319-30745467	GAC TCC CCC GAC TCA AAT CA	CCT TGA GCC CTG CAG TAG TT	/56-FAM/TGC ATG CTA /ZEN/CCA AGT CCT TGG C/3IABkFQ/
Gm13152_13154	chr4:146902213-146902348	GGT TTC TTG ATG CCA TTT CTG	CTA AAG AAT TCT CCA AAT ATT CCA GAT	/56-FAM/CGG GAC TGG /ZEN/TTG TGT GTG G/3IABkFQ/
Gm1995	chr12:89101101-89101237	GCA TCG TCC ACA GCT CAC T	TTT GGA CGA ACA GAA CTT GAG AAA	/56-FAM/CCG AAA GAA /ZEN/TTT GGC TGC AAG GTC /3IABkFQ/
Gm21671	chr5:26396876-26396971	TAT GGA TCT GGA CGT CTG CAT	GCA GTT CAG ATC CCT CAC TTC T	/56-FAM/AGT GGC AGT /ZEN/ATG TCC CGC TGC /3IABkFQ/
Gm7120	chr13:120281623-120281771	GGT TTC CCA TAA TTC AGC ACA A	CGC TAA AAA TCC CGC TAA AAG A	/56-FAM/AGT GAT CGG /ZEN/CAT GTG GTC GTG /3IABkFQ/
Gzma	chr13:113885136-113885241	AGG GTT TAA AAC TGT TGC CAA GTA	ACC CCA ATC AAA GAA TGG ATA AGC	/56-FAM/AGG CCC TGT /ZEN/TCG TCC TGT TTT G/3IABkFQ/
Hjurp	chr1:90162052-90162174	GCT GCG GTG ACA GAC AAT AC	TGT GGC CCA GTA CAG CTA TT	/56-FAM/CTT CCC ACA /ZEN/GCC TGG AGA GCC T/3IABkFQ/
Ifi27l2a	chr12:104680772-104680878	TCT ATA GCA GGA CAG AGG GGT A	GGT GCT GAA TGG CTA AAG TAG G	/56-FAM/TCA GAG CAC /ZEN/AAG TAG CAT GCC TCA /3IABkFQ/
Luzp4	chrX:145321717-145321816	ATC CCA GCG GTT GAA CTT TG	ATG CCT CCA AAC TCA AGT AAG A	/56-FAM/CTC GGG AGC /ZEN/AGA GTA GCA GGG /3IABkFQ/
Mup20	chr4:61676236-61676353	AAC TTT GGA TCT GGC TAA AAT CAG	AGA TAC TCA AGT TCT TCC ATA CTC GTA	/56-FAM/AGG GGG TGG /ZEN/GGA CTG AAC TG/3IABkFQ/
Nlrp1c-ps	chr11:71075987-71076085	GCA GAC ACG AGA AAG TTG AGT T	AGT GCT AGG ATC TGG TAT TGC A	/56-FAM/TAC AAA CCA /ZEN/AAA GGC TGG GAG CA/3IABkFQ/
Nxpe5	chr5:138690641-138690765	TTG GTG TTG TGT TTG AGG TAT TTG	GTA ACT AAA TAG GGG AAG CAA CTT G	/56-FAM/CTA ACC AGT /ZEN/GCA CAC TCC AGC CT/3IABkFQ/
Rsph1	chr17:31402067-31402192	GTT TGT TCT TGC CTG GAT TGT ATG	CTG GGT CAT TTG CAT GTT TGT AAC	/56-FAM/TAG AGC CAA /ZEN/GCC TCA GGG CG/3IABkFQ/
Sfi1	chr11:3084639-3084750	ATA GGT TTG GGC TGA GGA TAT GTA	AAC CCT TCT TAC TTG GTT CTT CAC	/56-FAM/ACA GTA GTG /ZEN/CAA AGC TGT CGG GTA G/3IABkFQ/
Smok2a	chr17:13418895-13418977	TGA TTT TGG ACT TGG CAT CC	CCT CAG GAG CAC TAA ATG GGT A	/56-FAM/CCA GGG CAA /ZEN/AAA CTA AAC TTA TTC TGT GG/3IABkFQ/
Tex24	chr8:28455579-28455682	TTG GAA AGA AGG CAC AAT GTC AAC	CAT CTC CAT AGC CTG CAT CCT	/56-FAM/AGC CTG GAG /ZEN/GTC CTC AAG AGG AG/3IABkFQ/
Tert	chr13:73765136-73765273	CCT CTG TGT CCG CTA GTT ACA	TCT TTG TAC CTC GAG ATG GCA	/5HEX/CCC GTG GGC /ZEN/AGG AAT TTC ACT A/3IABkFQ/

References cited in Suppl. Material

Abyzov A, Urban AE, Snyder M, Gerstein M. 2011. CNVnator: an approach to discover, genotype, and characterize typical and atypical CNVs from family and population genome sequencing. *Genome Res* **21**: 974-984.

Conrad DF, Andrews TD, Carter NP, Hurles ME, Pritchard JK. 2006. A high-resolution survey of deletion polymorphism in the human genome. *Nat Genet* **38**: 75-81.

Cooper GM, Nickerson DA, Eichler EE. 2007. Mutational and selective effects on copy-number variants in the human genome. *Nat Genet* **39**: S22-S29.

Egan CM, Sridhar S, Wigler M, Hall IM. 2007. Recurrent DNA copy number variation in the laboratory mouse. *Nat Genet* **39**: 1384-1389.

Keane TM, Goodstadt L, Danecek P, White MA, Wong K, Yalcin B, Heger A, Agam A, Slater G, Goodson M et al. 2011. Mouse genomic variation and its effect on phenotypes and gene regulation. *Nature* **477**: 289-294.

Li H, Handsaker B, Wysoker A, Fennell T, Ruan, Homer N, Marth G, Abecasis G, Durbin R. 2009. The Sequence Alignment/Map format and SAMtools. *Bioinformatics* **25**: 2078-2079.

Lin LI. 1989. A concordance correlation coefficient to evaluate reproducibility. Biometrics **45**: 255-268.

Magi A, Tattini L, Pippucci T, Torricelli F, Benelli M. 2012. Read count approach for DNA copy number variants detection. *Bioinformatics* **28**: 470-478.

Medvedev P, Stanciu M, Brudno M. 2009. Computational methods for discovering structural variation with next-generation sequencing. *Nat Methods* **6**: S13-20.

Quinlan AR, Hall IM. 2010. BEDTools: a flexible suite of utilities for comparing genomic features. *Bioinformatics* **26**: 841-842.

Raychaudhuri S, Korn JM, McCarroll SA, International Schizophrenia Consortium, Altshuler D, Sklar P, Purcell S, Daly MJ. 2010. Accurately assessing the risk of schizophrenia conferred by rare copy-number variation affecting genes with brain function. *PLoS Genet* **6**: e1001097.

Redon R, Ishikawa S, Fitch KR, Feuk L, Perry GH, Andrews TD, Fiegler H, Shapero MH, Carson AR, Chen W et al. 2006. Global variation in copy number in the human genome. *Nature* **444**: 444-454.

Sebat J, Lakshmi B, Troge J, Alexander J, Young J, Lundin P, Månér S, Massa H, Walker M, Chi M et al. 2004. Large-scale copy number polymorphism in the human genome. *Science* **305**: 525-528.

Sharp AJ, Locke DP, McGrath SD, Cheng Z, Bailey JA, Vallente RU, Pertz LM, Clark RA, Schwartz S, Segraves R et al. 2005. Segmental duplications and copy-number variation in the human genome. *Am J Hum Genet* **77**: 78-88.

Wong K, Bumpstead S, van der Weyden L, Reinholdt LG, Wilming LG, Adams DJ, Keane TM. 2012. Sequencing and characterization of the FVB/NJ mouse genome. *Genome Biol* **13**: R72.

Zhao M, Wang Q, Wang Q, Jia P, Zhao Z. 2013. Computational tools for copy number variation (CNV) detection using next-generation sequencing data: features and perspectives. *BMC Bioinformatics* **14**: S1.
